# Regulatory B Cells and Its Role in Central Nervous System Inflammatory Demyelinating Diseases

**DOI:** 10.3389/fimmu.2020.01884

**Published:** 2020-08-20

**Authors:** Zhou Ran, Luo Yue-Bei, Zeng Qiu-Ming, Yang Huan

**Affiliations:** Department of Neurology, Xiangya Hospital, Central South University, Changsha, China

**Keywords:** regulatory B cells, central nervous system, inflammatory demyelinating diseases, multiple sclerosis, neuromyelitis optica

## Abstract

Regulatory B (Breg) cells represent a population of suppressor B cells that participate in immunomodulatory processes and inhibition of excessive inflammation. The regulatory function of Breg cells have been demonstrated in mice and human with inflammatory diseases, cancer, after transplantation, and particularly in autoinflammatory disorders. In order to suppress inflammation, Breg cells produce anti-inflammatory mediators, induce death ligand-mediated apoptosis, and regulate many kinds of immune cells such as suppressing the proliferation and differentiation of effector T cell and increasing the number of regulatory T cells. Central nervous system Inflammatory demyelinating diseases (CNS IDDs) are a heterogeneous group of disorders, which occur against the background of an acute or chronic inflammatory process. With the advent of monoclonal antibodies directed against B cells, breakthroughs have been made in the treatment of CNS IDDs. Therefore, the number and function of B cells in IDDs have attracted attention. Meanwhile, increasing number of studies have confirmed that Breg cells play a role in alleviating autoimmune diseases, and treatment with Breg cells has also been proposed as a new therapeutic direction. In this review, we focus on the understanding of the development and function of Breg cells and on the diversification of Breg cells in CNS IDDs.

## Introduction

The immune response feedback is an important mechanism that maintains the immune balance. Inflammatory diseases such as systemic lupus erythematosus (SLE), rheumatoid arthritis (RA) and multiple sclerosis (MS) are hallmarks of immunologic imbalances. As a major component of the immune system, B cells play both positive and negative roles in innate and adaptive immunity, through effector molecules such as antibodies and cytokines as well as through antigen-presention. On the one hand, B cells can mediate several negative processes such as amplifying immune responses. Mechanistically, they differentiate into plasmablasts that secrete effector antibodies (1), may modulate effector T cell response through antigen presentation (2) and production of inflammatory cytokines (3). In addition, there is also a subset of B cells that regulates immune response to pathogens and autoantigens. These regulatory B cells are core targets in autoimmune and infectious diseases as well as cancer. These cells have a huge therapeutic potential against the aforementioned diseases. In one study performed in 1974 on delayed-type hypersensitivity, it was found that when B cells were removed, adoptively transferred splenocytes induced more intense reactions and lost their ability to suppress the delayed-type hypersensitivity reactions. This suggests that B cells or their products mediates inhibition of excessive inflammatory response (4). In another study conducted in 1996, it was found that mice with experimental autoimmune encephalomyelitis (EAE) but lacking B cells displayed greater differences in disease onset, severity, and recovery compared with the wild type group (5). Other studies on colitis and arthritis have demonstrated that B cells have antibody-independent immunoregulatory function (6, 7). Elsewhere, researchers have suggested that B cells inhibits excessive inflammation. B cells associated with inhibitory functions are referred to as Breg cells. IL-10 has been found to play a crucial role in the recovery of EAE (8). Other studies have further demonstrated that IL-10^−/−^ mice display a non-remitting course of EAE, similar to the B cell-deficient mice (9). Combined, these findings suggest that B cells regulatory functions are mediated by IL-10. B cell-derived IL-10 has indeed been shown to play a key role in controlling autoimmunity (10). Accordingly, expression of IL-10 has been widely used to define suppressive B cell populations in mice and humans (11). B cells also regulate inflammation by a variety of IL-10-independent mechanisms (12).

Central nervous system Inflammatory demyelinating diseases (CNS IDDs) is a term referring to several CNS disorders, characterized by damaged myelin sheath of neurons, thus impairing transmission of signal by affected nerves. CNS IDDs can be differentiated based on disease severity and temporal courses, imaging, laboratory test and pathological characteristics. CNS IDDs mainly include MS, neuromyelitis spectrum disorders (NMOSD), and myelin oligodendrocyte glycoprotein antibody-associated disease (MOG-Ab associated disease) (13). IDDs were considered to be primarily mediated by T lymphocytes. Given the success of therapeutic B cell depletion in MS (14) and NMOSD (15), there is growing concern on the role of B cells in the pathogenesis of IDDs. Studies on auto antibodies have improved our understanding of the role of B cells in the pathogenesis of immune-mediated diseases such as the appearance of oligoclonal IgG bands and deposition of IgG in the cerebrospinal fluid of MS, the presence of AQP4-IgG in NMOSD and antibodies against MOG in MOG-Ab associated disease. In addition, Breg cells also play a role in CNS IDDs. For instance, Breg cells deficiency is associated with severe symptoms of MS (16) and NMOSD (17), suggesting that Breg cells have the therapeutic potential to reduce immune-mediated inflammatory disorders. Subsequently, this review aimed at providing a summary of the current understanding on the development and function of Breg cells, and their role in the etiology of CNS IDDs.

## Development and Differentiation of Breg Cells

**Figure 1:**
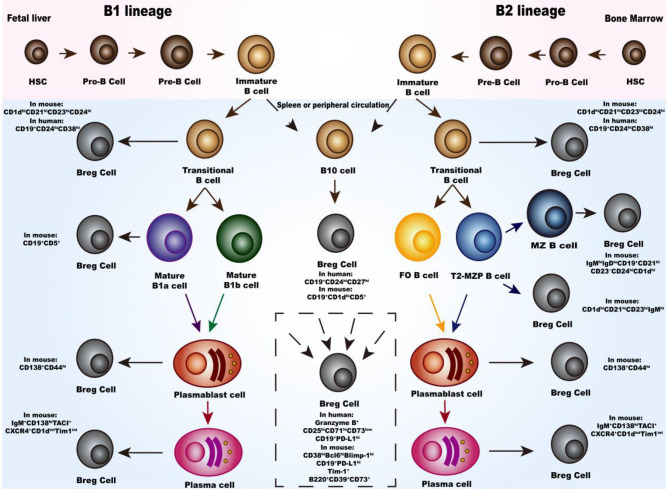
Development and differentiation of Breg cells. In bone marrow or fetal liver, hematopoietic stem cells (HSC) differentiate through progenitor B cells (Pro-B), precursor B cells (pre-B), immature B cells and transitional B cells. For the B1 subset, migrating to the spleen or peripheral circulation, the transitional B cells differentiate into either mature B1a cells or mature B1b cells and eventually differentiate into plasmablast cells and plasma cells. Meanwhile, for the B2 subset, the transitional B cells differentiate either into follicular (FO) or marginal zone (MZ) B cells and eventually into plasmablast cells and plasma cells. Moreover, there is the transitional-2 marginal-zone precursors (T2-MZPs) B cells stage which is the precursor of MZ B cells. Studies have proved that the transitional B cells, MZ cells, T2-MZP cells, B1 cells, plasmablasts, and plasma cells can all differentiate into Breg cells with different phenotypes. Differentiation pathway of B10 cells in the middle of the photograph are listed separately because there are still not enough studies to classify them. In the dotted box, there are Breg cells with different phenotype and the source of them are still not clear.

**Figure 2:**
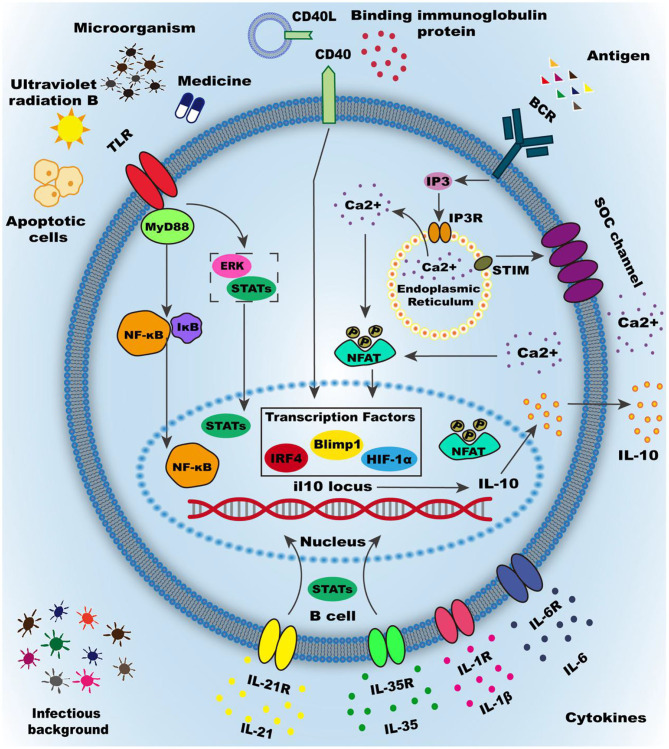
Activation of Breg cells. Numerous mechanisms have been demonstrated to induce Breg cells, the most important of which are summarized as the inflammatory micro-environment and intercellular interactions. The inflammatory microenvironment is mainly produced by inflammatory factors, such as IL-35, IL-21, and other inflammatory cytokines. Cytokines often mediate activation of downstream signaling pathways by binding to cytokine receptors on the membrane. Microorganisms infection is another essential part of the inflammatory environment in the body, including invasive bacteria and commensal microbiota. Most microbial infections are activated by downstream molecular pathways mediated by membrane surface molecules, causing IL-10 transcription and translation and secretion. Intercellular interactions play a vital role in the induction of regulatory B cells, such as apoptotic cells or type3 innate lymphoid cells. Intercellular interactions mainly depend on the interaction of molecules on the surface of the membrane and the most classic surface molecules are Toll-like receptors (TLRs), CD40, B cell receptor (BCR). Many environmental and pharmaceutical factors can activate TLRs and cause downstream pathways activation, such as STATs and ERK orMyD88-NF-κB signaling pathway. CD40 can be activated by the CD40 ligand or binding immunoglobulin protein to mediate the activation of downstream signaling pathways STATs, and the downstream signaling pathways can be enhanced through synergy with other cell membrane surface molecules. BCR is thought to be involved in multiple functional processes of B cells, especially the induction of Breg cells. BCR combined with antigen can promote the release of calcium ions into the cell from the endoplasmic reticulum (ER) and also promote the activation of STIM on the endoplasmic reticulum, which open the calcium ion channels (SOC channel) on the cell membrane. A large amount of calcium influx increases the intracellular calcium ion concentration and promotes the phosphorylation of the nuclear factor of activated T cells (NFAT) in the downstream pathway, thereby increasing the transcription and translation secretion of IL-10. Other membrane surface molecules can promote Breg cell differentiation, such as Galectin-1and CD38. Research on regulatory B cell-specific transcription factors is still inconclusive, but the transcription factors such as Blimp1, IRF4, andHIF-1α have been shown to promote the transcription of IL-10.

There are two distinct populations of B cells identified in mouse and human; the B1 and B2 subsets. Similar to other immune cells, B cells are derived from hematopoietic stem cells (HSCs), where they differentiate into progenitor B cells (Pro-B), precursor B cells (Pre-B) and immature B cells (Figure 1). Immature B cells undergo a “transitional” state, which is an early phase to the mature phenotype, after which they leave the bone marrow or fetal liver. B1 subset differentiates into mature B1a cells expressing CD5, and mature B1b cells. After stimulation with polysaccharides or lipids, mature B1 cells differentiate into antibody-secreting plasmablasts and short-lived plasma cells secreting antigen-specific antibodies. As for the B2 subset, they undergo three consecutive transitional B cells stages; transitional-1 (T1), transitional-2 (T2) and transitional-3 (T3). Transitional-B cells then migrates to the spleen and lymph node follicles, where they eventually differentiate into either follicular (FO) or marginal zone (MZ) B cells. The intermediate subset between T-2 B and MZ B cells are transitional-2 marginal-zone precursors (T2-MZP) B cells. Activated MZ B and FO B cells eventually differentiate into plasma cells, antibody producing B cells. Under special conditions, transitional B cells, MZ cells, T2-MZP cells, B1 cells, plasmablasts and plasma cells can all be activated to differentiate into Breg cells. Inflammatory microenvironment and intercellular interaction have been identified to activate this differentiation. Details of these processses will be discussed in the subsequent sections (Figure 2).

### Inflammatory Microenvironment

The inflammatory microenvironment such as infiltration of cytokines and infection microenvironment can increase the expression as well as enhance the inhibitory property of Breg cells, implying that such factors play important roles in differentiation of Breg cells.

Numerous studies show that most inflammatory cytokines can indeed induce differentiation of Breg cells. As an immunosuppressive heterodimeric cytokine, IL-35 binds on its corresponding IL-35 receptor, activating signal transducer and activator of transcription (STAT) 1 and 3 to induce differentiation of resting B cells into IL-10 and IL-35-producing Breg cells. This suggests that IL-35 has the potential to induce autologous Breg cells as well as the treatment of autoimmune and inflammatory diseases (18, 19). More studies have demonstrated that IL-10-producing dendritic cells induced by IL-35 and phosphorylating STAT3 can induce immunosuppressive property of IL-10-producing B cells (20).

Similar to IL-35, IL-21 also induces production of IL-10 via phosphorylating STAT3. Accordingly, inhibition of phosphorylating STAT3 effectively blocks the production of IL-10 during the differentiation of the Breg cells. The effect of IL-21 on the differentiation of Breg cells strongly depends on additional signals including inhibitors of Toll-like receptors (TLR) and simulation of both B-cell receptor (BCR) stimulation and CD40 ligand. For instance, with the help of CD154 (CD40 ligand), IL-21 induces the differentiation of B cells into plasma cells or granzyme^+^ B lymphocytes (an important type of Breg cells) (21). In addition, the maturation of Breg cells into effector cells that secrete functional IL-10 requires homologous interactions with T cells mediated by IL-21 and CD40 (22).

Other inflammatory cytokines critical for differentiation of Breg cells include IL-1β, IL-6 and granulocyte-macrophage colony-stimulating factor (GM-CSF). In mice with arthritis, deficiency of B cell specific IL-6 or IL-1 receptors is shown to exacerbate the disease compared with the controls (23). GM-CSF and IL-15 are strong immunosuppressive molecules that can induce differentiation of naive CD19^+^ B cells into Breg cells, a process that can reverse the neuropathology of EAE (24).

Surprisingly, immune response to infectious diseases does not always worsen autoimmune diseases. In some instances, response to infectious diseases drives the development of IL-10-producing Breg cells in both mice and humans. The helminth parasite *Schistosoma mansoni* contains TLR4 inhibitor, thus is able to induce secretion of IL-10 by B cells. This can then change the course of MS and reduce the severity of the disease (25). Similarly, *Mycobacterium tuberculosis* contains a TLR inhibitor, thus infection by this bacteria can aid in the recovery of EAE because it mediates the production of IL-10 by B cells. In a clinical trial, Bacillus Calmette-Guerin (BCG), a vaccine against tuberculosis disease, has been shown to alleviate clinically isolated syndrome (CIS) by reducing the number of lesions and improving long-term disease course (26). In MS, the severity of the disease significantly decreased after the reception with BCG vaccine (27). The underlying infection is not limited to invasive bacteria, but also includes the commensal microbiota in the intestines. These microorganisms have been shown to promote the differentiation of Breg cells in mesenteric lymph nodes and the spleen (23).

### Intercellular Interaction

Intercellular interaction can also induce the differentiation of primary B cells into Breg cells, mainly through the activation of surface molecules on B cells (such as TLRs, CD40, BCR) and subsequent B cell downstream signaling pathway.

Gray M et al. found that apoptotic cells (ACs) affects the production of IL-10. This was demonstrated by injection of ACs into collagen-induced arthritis model, which induced the production of IL-10 by Breg cells, a process that alleviates inflammation (28). Gray M et al. also demonstrated the mechanism underlying secretion of IL-10 by B cells. Here, after recognizing the DNA containing complex on the surface of ACs, naturally occurring B cells (such as MZ B cells) bind and internalize the ACs surface chromatin complex, thereby activating TLR9 to regulate proliferation of B cells and secretion of IL-10 (29). Type 3 innate lymphoid cells (ILC3s) and innate B cells interact through IL-15 and B cell activating factors (BAFF), a process that promotes the development of ILC3s with CD40 ligand. CD40 positive ILC3s aid in the proliferation and differentiation of IL-10-secreting B cells. This mutually beneficial relationship between cells is important for maintaining immune tolerance, however, there are several deficiencies in this relationship in allergic asthmatic patients (30). By releasing IFN-α that interacts with CD40, dendritic cells can also drive the differentiation of immature B cells into IL-10-producing Breg cells. Conversely, Breg cells inhibits production of IFN-α by dendritic cells mediated by IL-10. In SLE, there are defects in this cross-talk, believed to be associated with abnormal activation of STAT1 and STAT3 (31).

TLRs are necessary for B cells to exert their inhibitory effects such as inhibition of inflammatory T cell responses and modulation of inflammation. TLRs-myeloid differentiation factor88 (MyD88) pathway is closely associated with the anti-inflammatory immune mechanism. In mouse and human, the activation of TLR2, TLR4, and TLR9 transduction signal can induce production of IL-10 in B cells. For instance, trametes versicolor is a medicinal fungus that can promote differentiation of B cells into CD1d^+^ Breg cells in acute colitis, through the TLR2/4-mediated signaling pathway (32). Apart from chemical means, physical activation of B cells by factors such as ultraviolet radiation B has also been shown to induce differentiation of B cells into Breg cells. This process also suppresses the immune response through the TLR4-mediated signaling pathway (33). However, not all TLR stimulation can induce B cells to differentiate into Breg cells. For example, after activation through TLR7 and interferon-α, transitional B cells develop into pathogenic B cells, promoting the production of autoreactive antibodies (34). Studies on downstream mechanisms found that IFN-α can differentially regulate TLR7/8 and TLR9-activated STAT3 and ERK in B cells (35). More specifically, stimulation of B cells mediated by IFN-α and TLR7/8 inhibitors enhances phosphorylation of ERK1/2 and STAT3, which intern mediated production of IL-10 by B cells. Moreover, the activation of ERK and STAT3 is also important in TLR9- mediated IL-10 producing by B cells. However, IFN-α is not able to enhance the CpG-induced phosphorylation of ERK1/2 and STAT3 in B cells. MyD88 is a key downstream molecule in the inflammatory signaling pathway that also plays an important role in the regulation of cellular mediated immunity during infection (36). For instance, during *Helicobacter felis* infection, B cells activated by Helicobacter TLR-2 ligands can actuate IL-10-producing B cells in a MyD88 dependent manner (37). Endogenous TLR4 ligands are also found to be up-regulated, and activate B cells to produce IL-10 via TLR4-MyD88 signaling (38, 39). At transcription level, NF-κB plays an essential role in the inflammatory and immune response of cells, and the mis-regulation of NF-κB may cause autoimmune diseases, chronic inflammation and many types of cancer. Most importantly, with infectious diseases, activation of TLRs-MyD88-NF-κB can induce production of B cells specific IL-10 (40). On tumor research, one study found that activation of the TLRs-MyD88-NF-κB signaling pathway is necessary for Breg cells differentiation and the induced Breg cells with immunoregulatory functions can contribute to the suppression of anti-tumor immunity (41). This aside, IκBNS is a TLR-inducible nuclear IκB protein important in the TLRs-mediated IL-10 production in B cells. Mechanistically, IκBNS regulates inflammatory responses by inhibiting the induction of a subset of TLR-dependent genes through modulation of NF-κB activity (42). IκBNS-deficient B cells show reduced expression of Breg cells transcription factors including B lymphocyte induced maturation protein 1 (Blimp-1 protein) and interferon regulatory factor 4 (IRF4). They also fail to generate IL-10 producing CD138^+^ plasmablasts (a subset of Breg cells), suggesting that IκBNS is selectively required for IL-10 production in B cells, responding to TLR signals (43).

CD40, a membrane-associated protein, is a member of the tumor necrosis factor (TNF) receptor superfamily. The activation of CD40 on B cells not only induces the maturation of B cells into antibody-producing B cells, but is also crucial for the activation of Breg cells. In transgenic mice, ectopic expression of the CD154 is associated with increased CD40 signaling, which can in turn induce activation of the STAT3 pathway and an increase in the proportion of Breg cells (44). In experimental lupus, stimulation with CD40 inhibitor can induce IL-10-producing T2-like B cells to suppress Th1 responses and induce suppressive capacity to CD4^+^ T cells. This reduces the severity of the disease and delays IL-10 dependent progression of the disease (45). Furthermore, the synergistic effect of cell surface membrane molecules can activate B cells to perform their regulatory functions. For instance, co-stimulation of CD40 and TLRs has been shown to induce the highest proportion of IL-10 producing Breg cells, which plays a crucial role in recovery from MS relapse (46). Besides, an immunoglobulin protein (BIP), a member of the heat shock protein 70 family, can act in synergy with CD40 to induce the differentiation of Breg cells as well as suppress proliferation of T cells in a partially IL-10-dependent manner (47).

The BCR-STIM pathway is involved in most B cell function processes, such as activation, differentiation and antigen recognition, endocytosis, and presentation. BCR-stimulated B cells can maintain long-term tolerance and protect vulnerable mice from type 1 diabetes via an IL-10-dependent mechanism (48). At transcription level, many regulatory molecules are involved in IL-10 expression, including stromal interacting molecules (STIM) of endoplasmic reticulum (ER), nuclear factor of activated T cells (NFAT) family, IRF4 and a crucial cis-regulatory element CNS-9 that is located 9kb upstream of the transcription start site of *il10*. After BCR activation, calcium sensory proteins STIM-1 and STIM-2 induce store-operated Ca^2+^ (SOC) influx and proliferation to increase the intracellular calcium concentration. This can activate the signaling pathway of NFAT family and mediate secretion of IL-10 (49). Without STIM1 or STIM2 proteins, B cells fail to produce IL-10 due to the defect in the activation of NFAT after BCR stimulation. Besides, the CNS-9 region contains clusters of NFAT and IRF binding motifs, which enhances the expression of IL-10 mRNA through the synergy effect of NFAT1 and IRF4. Therefore, deficiency of Irf4 specific to B cells impairs secretion of IL-10 and abnormal differentiation of Breg cell in dLNs. This intern predispose one to EAE (50).

Some other surface molecules such as CD38, Galectin-1 (Gal-1). may also play a role in the expression and function of Breg cells. For instance, CD38 is a transmembrane protein expressed in B lymphocytes. It can induce proliferation, differentiation or apoptosis of Breg cells. Currently, studies have found that the expression and effect of CD38 are inconsistent in different diseases. Some studies have demonstrated that CD38^−/−^ mice are more suitable for generating and expanding regulatory B10 cells than WT mice under appropriate stimulation (51). However, other studies have suggested that CD1d^hi^CD5^+^ Breg cells highly expresses CD38, and in the presence of a CD38 stimulator, the percentage of Breg cells and their IL-10 production function increases (52). Galectin-1 (Gal-1) is a class of protein necessary for B cell development in the bone marrow. It plays a role in inducing B cell regulatory functions. Compared with wild-type B cells, Gal-1^−/−^ B cells have impaired IL-10 and Tim-1 expression, but with increased expression of TNF-α (53).

## Phenotypes of Breg Cells

The activation process of immune cells is different in various disease states. Therefore, many surface markers used to identify Breg cells are either up- or down-regulated, which results in the non-uniform molecular expression of Breg cells. Studies in experimental animal models as well as in patients with autoimmune diseases have identified multiple subsets of Breg cells that exhibits diverse immune suppression mechanisms (see Tables 1, 2 for the function of various Breg cell subtypes in mice and humans). However, due to the intricate origins and activation pathways of Breg cells, there is an ever-increasing list of new phenotypic and functional markers associated with Breg cells.

**Table 1:** The phenotype and function of mouse regulatory B cell subsets.

**Name**	**Phenotype**	**Functions**
Transitional2 B cells	CD1d^hi^CD21^hi^CD23^hi^CD24^hi^	1. Supress the Th1 responses (45)
		2. Inhibit T cell cytokine production to prolong graft survival (54)
MZ B cells	IgM^hi^IgD^lo^CD19^+^CD21^hi^CD23^−^CD24^hi^CD1d^hi^	1. Suppress CD8 and CD4 effector functions (55)
T2-MZP B cells*	CD1d^hi^CD21^hi^CD23^hi^IgM^hi^	1. Inhibit inflammatory cytokines production, suppress Ag-specific T cell activation and reduce in cells exhibiting Th1-type functional responses (56)
		2. Regulate both CD4^+^CD25^−^ T cells and regulatory T cells (57)
		3. Promote tumor cell growth (58)
B10 cells	CD19^+^CD1d^hi^CD5^+^	1. Suppress proliferation and differentiation of Th17 cells (59)
		2. Inhibit the production of Th1 cytokines, regulate the Th1/Th2 balance and maintain Treg cells (60)
		3. Inhibit microglial responses (61)
		4. Promote tumor cell proliferation (62)
B1a cells	CD19^+^CD5^+^	1. Inhibit macrophage proinflammatory responses and promote Treg cell responses (63)
		2. Regulate neutrophil infiltration, CD4^+^ T cell activation and proinflammatory cytokine production (64)
		3. Inhibit proinflammatory cytokine secretion, mediate CD4^+^ T cell apoptosis and prevent inflammation progressing (65)
		4. Negatively regulate anti-tumor immunity (66)
Plasmablast	CD138^+^CD44^hi^	1. Limit autoimmune inflammation and reduce disease severity (50)
Plasma B cells	IgM^+^CD138^hi^TACI^+^CXCR4^+^CD1d^int^Tim1^int^	1. Induce the Treg cell-mediated suppression (67)
		2. Activate macrophages to produce cytokines that reduce the anti-tumor immune response (68)
PD-L1^hi^ B cells	CD19^+^PD-L1^hi^	1. Mediate the generation of regulatory T cells (69)
TIM-1^+^ B cells	TIM-1^+^	1. Promote Th2 responses and regulate immune tolerance (70)
		2. Promote the generation of regulatory T cells (71)
CD39^+^CD73^+^B cells	B220^+^CD39^+^CD73^+^	1. Suppress effector T cell functions (72)

* *It is difficult to find a unified phenotype of T2 and T2-MZP B cells, so there are the most common and recognized cell phenotype of these two cells*.

**Table 2:** The phenotype and function of human regulatory B cell subsets.

**Name**	**Phenotype**	**Functions**
Immature/Transitional B cells	CD19^+^CD24^hi^CD38^hi^	1. Suppress effector T cell but enhance Treg cell functions (44, 73)
Memory B cells/B10 cells	CD19^+^CD24^hi^CD27^+^	1. Induce Foxp3 expression on regulatory T cells (74)
		2. Have the value of evaluating the efficacy of biological drugs (75)
GrB^+^ B cells	IgM^+^CD19^+^CD38^+^CD1d^+^CD147^+^	1. Inversely related to disease activity and clinical characteristics (76)
		2. Negatively modulate Th1 and Th17 cells, induce T cell apoptosis and strongly suppress T cell proliferation (77)
		3. Suppress antitumor immune responses (78)
Br1 cells	CD25^hi^CD71^hi^CD73^low^	1. Suppress antigen-specific CD4^+^ T cell proliferation (79)
PD-L1^hi^ B cells	PD-L1^+^	1. Suppress pro-inflammatory cytokine production (80)
		2. Exhibit T cell suppressive capacity (69)
		3. Repress the proliferation and activation of CD8^+^ T cells (81)

### Breg Cells in Mouse

#### Transitional-2 B Cells (CD1d^hi^CD21^hi^CD23^hi^CD24^hi^)

CD1d molecules are cell surface glycoproteins and the cytoplasmic tail of CD1d participates in signaling cascades associated with the transcription of IL-10 (82). In autoimmune diseases, the induced B-cell subpopulation is characterized by CD1d up-regulation, and the up-regulated CD1d can induce B-cell subpopulations to produce IL-10, promote antigen-specific regulatory T cell differentiation, and down-regulate inflammatory cascades associated with IL-1 upregulation and STAT3 activation (6). CD1d is expressed on a wide variety of cell types, of which three different B cell subsets express high levels of CD1d and have the potential to become Breg cells, including T2 B cells, MZ B cells and T2-MZP B cells. In the early stages of differentiation, B cells have already had the ability to differentiate into Breg cells. In SLE model, the adoptive transfer of T2 B cells can reverse autoimmunity and suppress the Th1 response (45). CD40 ligation halts the apoptosis of T2 B cells and prevents further differentiation into mature FO B cells (83), which can induce and expand the differentiation of IL-10^+^ T2 B cells. In allograft rejection model, T2 B cells isolated from tolerant mice show higher survival rates and inhibit cytokine production of T cells, thereby prolonging graft survival, suggesting that T2 B cells have the potential to treat allograft rejection (54).

#### MZ B Cells (IgM^hi^IgD^lo^CD19^+^CD21^hi^CD23^−^CD24^hi^CD1d^hi^)

MZ B cells with CD1d high expression play a role in the prevention of autoimmunity through the production of regulatory cytokines and natural antibodies. Besides the polyreactive BCRs, MZ B cells also express high levels of TLRs, such as TLR9 which recognizes hypomethylated CpG motifs in bacterial DNA or chromatin complexes expressed on the surface of apoptotic cells (29). MZ B cells can differentiate into IL-10-producing B cells and down-regulate the production of pro-inflammatory cytokines in response to stimulation of ligands or cytokines such as BAFF (84). Studies have demonstrated that through inflammatory stimuli, T-bet-expressing MZ B cells secrete IL-10, suggesting that T-bet might contribute to the remission of autoimmune diseases by activating the regulatory potential of MZ B cells (85). In collagen-induced arthritis model, MZ B cells produce most of IL-10 in response to TLR stimulation or apoptotic cells, and the adoptive transfer of MZ B cells could protect mice from infection (28). Study of Leishmania donovani infection have found that MZ B cells can interact with parasites to secrete IL-10 in a MyD88-dependent manner, and MZ B cells are involved in the suppression of CD8 and CD4 effector functions (55).

#### T2-MZP B Cells (CD1d^hi^CD21^hi^CD23^hi^IgM^hi^)

Compared with MZ B cells, T2-MZP B cells also express highly CD1d but produce IL-10 in much larger quantities. It is difficult to find a unified phenotype of T2 and T2-MZP B cells, so the distinction between these two cells is worthy of further research and discussion. The immunomodulatory effects of T2-MZP B cells in a variety of immune-mediated pathologies including autoimmune diseases, allergy diseases and cancer. The regulatory function of T2-MZP B cells has been first demonstrated in experimental arthritis model, and the realization of its regulatory effect depends on IL-10 mediation, inhibition the production of inflammatory cytokines, suppression of Ag-specific T cell activation, and reduction of Th1-type functional responses (56). Moreover, the regulatory effect of T2-MZP B cells can ameliorate the cellular infiltrates and the inflammatory damage by increasing Foxp3^+^ Treg cells and reducing the number of Th1 and Th17 cells (57). In Helicobacter felis infection model, T2-MZP B cells can induce the differentiation of T cells into a regulatory phenotype to ameliorate the inflammatory damage (37). In melanoma model, tumors initially signal via the lymphatic drainage to stimulate the preferential accumulation of T2-MZP Breg cells and this local response may be an early and critical step in generating an immunosuppressive environment to permit tumor growth and metastasis, suggesting T2-MZP B cells can promote tumor growth (58).

#### B10 (CD19^+^CD1d^hi^CD5^+^)

With the continuous expansion of the research scope, the exposure of B cells to different inflammatory environments had limited the use of CD1d markers to identify Breg cells. The co-expression of CD1d and CD5 on B10 cells has been therefore used to characterize the spleen B cell population. In mice, although B10 cells only account for about 1–2% of spleen B cells and 7–8% of peritoneal B cells, they are the main source of IL-10 production. Similar to B1a cells, MZ B cells and T2-MZP B cells, B10 is able secrete a large amount of IL-10 and express similar surface markers such as CD19, CD1d, CD21, and CD24. However, each Breg cell type may have different stimulatory requirements for IL-10 production. *In vitro*, B10 cells stimulated via the TLR2 and TLR4 latter express cytoplasmic IL-10 at hour 5. Studies show that B10 cells have a regulatory function in suppressing immune responses such as IL-10-dependent regulation of T cell-dependent autoimmune responses (11). The adoptive transfer of B10 cells can suppress proliferation and differentiation of Th17 cells via the reduction of phosphorylating STAT3 and expression of retinoid-related orphan receptorγt (RORγt). This cascade of events delays the onset of inflammation and reduces clinical symptoms and inflammatory damage (59). In silicosis, a disease characterized by chronic lung inflammation and fibrosis, B10 can inhibit the production of Th1 cytokines, regulate the Th1/Th2 balance and maintain Treg cells (60). In viral infection, B10 cells can infiltrate the chronically infected brains and inhibit the microglial response (61). Generally, B10 cells are potent negative regulators of antigen-specific inflammation and T-cell-dependent autoimmune diseases. Therefore, the reinfusion of B10 cells to control disease progression may provide an effective treatment for both inflammatory and autoimmune conditions. B10 cells play a pro-tumorigenic role by promoting tumor cell proliferation. In pancreatic cancer, a recent research found that the bruton's tyrosine kinase signaling pathway can play a role in regulating differentiation of B10 cells, thereby controlling the cancer (62).

#### B1a Cells (CD19^+^CD5^+^)

B1a cells are another major source of IL-10, inhibiting the progression of both innate and adaptive immune responses, but at the cost of impeding pathogen clearance. The tissue-specific signals and unique pathogen-derived signals combine to determine whether the response of B1a cells is predominantly regulatory or proinflammatory. Gray M et al. found that in response to ACs, B1a cells can inhibit macrophage proinflammatory responses and promote Treg cell responses to self-antigens in an IL-10 dependent manner (63). In colitis model, IL-10 production by B1a significantly reduced disease severity by regulating neutrophil infiltration, CD4^+^ T cell activation, and proinflammatory cytokine production during disease onset (64). In collagen-induced arthritis model, IL-10 produced by B1a cells inhibits proinflammatory cytokines secreted by activated macrophages and T cells in infectious lesions, and expressing Fas ligand (FasL) B1a cells can mediate CD4^+^ T cell apoptosis and prevent inflammation progressing (65). In addition to secreting IL-10, in controlling immune homeostasis, B1a cells can also convert naive T cells into T cells with regulatory activity through cell-to-cell contact (86). In melanoma tumor immunity, B1a cells negatively regulate anti-tumor immunity by producing IL-10, suggesting they can be a target for immunotherapy of tumor (66).

#### Plasmablast (CD138^+^CD44^hi^) and Plasma B Cells (IgM^+^CD138^hi^TACI^+^CXCR4^+^CD1d^int^Tim1^int^)

Studies have shown that in later stages of B-cell development such as plasmablasts and plasma cells can also produce IL-10 and have the inhibitory capacity. In autoimmune diseases, plasmablasts in the dLNs serve as IL-10 producers to limit autoimmune inflammation, while the absence of IL-10^+^ plasmablasts increases disease severity (50). In *Salmonella Typhimurium* infection, B cell-specific MyD88 signaling is essential for optimal development of IL-10-producing CD19^+^CD138^+^ B cells, especially in early stages of infection, and via MyD88 signaling, CD19^+^CD138^+^ B cells inhibit three key types of cells: neutrophils, natural killer cells, and inflammatory T cells (36). Similarly, plasma cells are also the major source of B-cell-derived IL-10 and IL-35 which can induce the Treg cell-mediated suppression (67). Plasma cells are found in the CNS of MS patients and the expression of IL-10 by plasma cells was necessary and sufficient to confer resistance toward inflammation, suggesting that plasma cells play an unexpected role in suppressing neuroinflammation (87). In hepatoma model, IgG-producing plasma cells activate macrophages to produce cytokines that reduce the anti-tumor immune response, while depletion of these plasma cells is able to prevent generation of activated macrophages, increase the anti-tumor T cell response, and reduce growth of tumor (68). As an essential regulator of plasma cell development, Prdm1 (encoding the Blimp-1 protein) is strongly correlated with IL-10 production, and during the formation of plasma cells, the prolonged elevation of Blimp-1 expression can elicit IL-10 production (88). Simon Fillatreau et al. identified a natural plasma cell subset characterized by the expression of the inhibitory receptor LAG-3, CD200, PD-L1, as well as PD-L2. Via a TLR-driven mechanism, natural regulatory plasma cells upregulate IL-10 expression within hours and without proliferating, suggesting that this group of plasma cells may be a potential disease treatment (89).

#### Other Breg Subsets

Programmed death ligand-1 (PD-L1) is important in controlling immune function, and promoting the proliferation of antigen-specific T cells. Besides this, programmed cell death receptor-1 (PD-1) binds to PD-L1 thereby transmitting inhibitory signals that reduce T cell proliferation. In RA, PD-L1^hi^ B cells can suppress disease development by elevating the expression of PD-L1. Presence of PD-L1 on B cells is positively correlated with Treg cells but negatively correlated with effector T cells, implying that PD-L1 mediates the generation of Tregs, an important molecule on B cells (69). T cell immunoglobulin mucin domain-1 (Tim)-1 is a membrane surface glycoprotein mainly expressed on cells, and is associated with regulation of immune responses. Apart from an inclusive marker for IL-10^+^ Breg cells derived from T2-MZP B cells, B10 cells and CD138^+^ B cells (90), Tim-1 is also critical for the induction and maintenance of Breg cells. Co-stimulation of IL-21, anti-Tim1 and CD40L can induce IL-10 activity in B10 cells and inhibit the progression of experimental periodontitis (91). TIM-1^+^ B cells strongly express IL-4 and IL-10, and promote Th2 responses, which can directly regulate immune tolerance (70). Conversely, B cells with Tim-1 defects are unable to produce IL-10 in response to ACs or by specific ligation with anti-TIM-1, or are unable to increase production of proinflammatory cytokine such as IL-1 and IL-6. This effect promotes Th1 and Th17 responses. In addition, B cells with defective Tim-1 can inhibit the generation of regulatory T cells and enhance the severity of autoimmune diseases (71). Collectively, these studies suggest that TIM-1 is critical in both the maintenance and induction of Breg cells under varied physiological conditions. CD39 and CD73 on their part are two ectoenzymes that together catalyze the dephosphorylation of adenine nucleotides to adenosine. Adenosine is known to suppress effector T cell function by binding on several adenosine receptors. Circulating B220^+^CD39^+^CD73^+^ B cells can drive a shift from an ATP-driven pro-inflammatory environment to an anti-inflammatory milieu induced by adenosine (72). A recent research found that decreased CD73 expression and the adoptive transfer of CD73^+^ B cells can impair production of adenosine, which can reduce the severity of colitis. This implies that CD39^+^CD73^+^ B cell adenosine can regulate autoimmune inflammation (92).

### Breg Cells in Humans

In humans, Breg cells maintains immune homeostasis. Breg cells in both human and mice are predominantly identified based on their IL-10 producing property.

#### Immature/Transitional B Cells (CD19^+^CD24^hi^CD38^hi^)

Previous studies have demonstrated that transitional B cells can exert IL-10 mediated inhibition of the expression of IFN-γ and TNF-α in T cells (44). In healthy individuals, transitional B cells suppress proliferation of CD4^+^ T cell as well as release of pro-inflammatory cytokines, a function that partially mediated via the production of IL-10. However, in various autoimmune diseases such as primary Sjögren's syndrome (93) and diabetic nephropathy (94), there is an under production and defective functioning of transitional B cells, particularly during the active phase of the disease. In autoimmune diseases, defective transitional B cells have impaired IL-10 production upon activation via the CD40. Due to a defect in STAT3 phosphorylation (44), Breg cells are unable to suppress Th1 responses and fail to mediate differentiation of CD4^+^T cells into functionally suppressive Treg cells. This suggests that Breg cells may fail to prevent the development of autoreactive responses and inflammation. The higher number of transitional B cells in patients receiving rituximab is associated with long-term remissions, suggesting that the re-aggregation of Breg cells may be associated with better disease outcome (95). Apart from autoimmunity, transitional B cells have been shown to play a key role in establishing transplant tolerance (96). Breg cells can inhibit effector T cell function during the immune response in transplantation (73). In infectious diseases such as with viruses, enhanced production of IL-10-producing transitional B cells positively correlates with the viral load. The Breg cells were also shown to suppress virus-specific CD8^+^ T cell responses but enhances function of regulatory T-cells via the production of IL-10 and possibly expression of PD-L1. This suggests that transitional B cells may contribute to immune dysfunction in virus infection, which can hinder the elimination of the infection (97).

#### Memory B Cells/B10 Cells (CD19^+^CD24^hi^CD27^+^)

Both memory B cells (also known as B10 cells in human) and transitional B cells are the major IL-10-producing B cells. They have similar functional characteristics such as suppressing proliferation of CD4^+^ T cells and inhibiting expression of pro-inflammatory cytokines. However, compared with transitional B cells, B10 cells have higher growth factor β (TGF-β) and shows stronger expression of granzyme B. B10 cells also express higher levels of surface integrins and CD39, suggesting the two Breg subsets have distinct functional characteristics (98). B10 cell subset additionally expresses high levels of TLR9, a receptor shown to be more sensitive to stimulation by CpG oligonucleotides. A proliferation-inducing ligand (APRIL) can stimulate signaling pathways activated by CpG (ERK1/2 and STAT3), which can induce an increase in the production of B10. This promotes production of IL-10 and induce expression of Foxp3 on regulatory T cells (74). Moreover, it is reported that miRNA-155 can regulate IL-10-Producing B10 cells in human by enhancing the expression of *il10* gene (99). Although B10 cells play a suppressive role, their function is altered differently in several autoimmune diseases such as Bullous pemphigoid (100). Moreover, B10 cells increases in RA patients treated with biopharmaceuticals, suggesting that B10 cells may represent a predictive biomarker for response to the treatment (75). In transplant-related diseases, the number of IL-10-producing CD24^hi^CD27^+^ B cells decreases. The function of the same cells is also impaired in graft-versus-host disease (cGVHD) (101). Similarly, in liver transplantation, patients that suffered from acute allograft rejection had significantly decreased proportions of B10 cells, but they dramatically increased after anti-rejection therapies (102).

#### Granzyme B^+^ B Cells (IgM^+^CD19^+^CD38^+^CD1d^+^CD147^+^)

Granzyme B(GrB) is a serine protease with several functions including antigen processing, matrix degradation, activation of inflammatory cytokines and immunoregulatory effects. GrB is dramatically elevated in chronic and inflammatory disorders. Secretion of GrB by B cells may play a significant role in early antiviral immune responses, regulation of autoimmune responses and in cancer immuno-surveillance. Studies show that co-stimulation of cytokines such as IL-21 and membrane surface molecules such as BCR, CD40 and TLRs induces B cells to differentiate into active forms that secrete cytotoxic serine protease (103). The immunoregulatory function of activated GrB^+^ B cells has been demonstrated in many human autoimmune diseases, suggesting that the impairment of this Breg cells subset is related to the pathogenesis of diseases (76). In RA, GrB-producing Breg cells are significantly decreased, and their proportion is negatively correlated with disease activity and clinical features. Moreover, the optimual level of these cells can be restored after effective therapy (104). Specifically, GrB^+^ B cells negatively modulate Th1 and Th17 cells, induce T cell apoptosis and strongly suppress T cell proliferation by downregulating the T cell receptor (TCR) zeta chain (77). Similar findings have been reported in tumor research. Other studies found that GrB^+^ B cells infiltrate tumor microenvironment and tumor-draining lymph nodes where they may participate in the suppression of antitumor immune responses (78). In transplant-related diseases such as in renal transplantation, the affected patients showed a diminished level of GrB^+^ B cells compared to healthy controls (105). In general, GrB^+^ B cells may participate in early cell-mediated immune responses during inflammatory and neoplastic processes. Therefore, a better understanding of the role of GrB-secreting B cells in the immune system may help develop and improve new immunotherapy methods for infectious, autoimmune and malignant diseases.

#### Other Breg Subsets

Type 1 regulatory B (Br1) cells are characterized by CD25^hi^CD71^hi^CD73^low^. They maintain peripheral blood tolerance by producing IgG4 antibodies (106). After receiving allergen-specific immunotherpay, the proportion of BR1 cells increases, secreting high levels of IL-10 (107). In autoimmune diseases, the function of Br1 is impaired while the inhibition of Th2 response islimited, implying that Br1 plays an important role in tolerance induction (79). Many Breg subtypes such as PD-L1^hi^ B cells are similar in both humans and mice. B cells can modulate T cell immune responses through the expression of regulatory molecules such as PD-L1. In autoimmune diseases, CpG induces expression of PD-L1 on human B cells, which suppresses pro-inflammatory cytokines produced from antigen-stimulated CD4^+^ T cells (80). PD-L1^hi^ B cells exhibiting T cell suppressive capacity are significantly decreased in untreated RA patients but normalize upon successful treatment (69). Besides, Tumor-infiltrating B cells that express high levels of PD-L1, IL-10 and TGF-β repress the proliferation and activation of CD8^+^ T cells (81).

## Breg Cell Effector Functions

**Figure 3:**
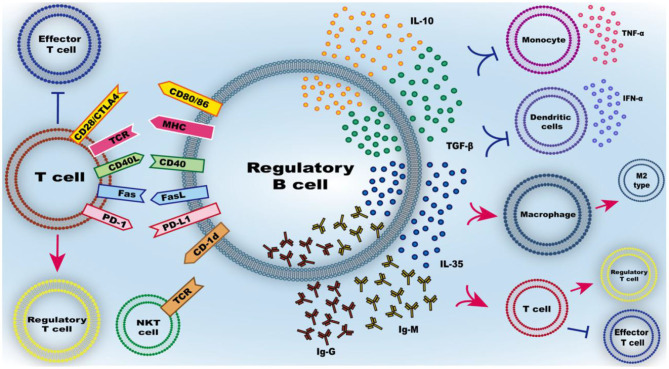
Function of Breg cells. The mechanism of Breg cell inhibitory effect mainly includes the secretion of inhibitory mediators and the inhibitory effect of intercellular contact. IL-10, as the main secreted inhibitory cytokine of regulatory B cells, has a variety of effects such as inhibiting the release of pro-inflammatory cytokines from immune cells, inhibiting the inflammatory differentiation of macrophage/microglia and promoting the conversion of T cells to regulatory T cells rather than effector T cells. Similarly, TGF-β, IL-35, IgG, and IgM can also regulate the differentiation of T cells into regulatory T cells. Membrane-bound molecules at the interface between regulatory B cells and T cells include CD80/86, CD40, MHC, FasL, PD-L1, and CD-1d, can regulate the differentiation of T cells into regulatory T cells and activate natural killer T (NKT) cells with suppressive function.

Breg cells identified in both mice and human have been shown to downregulate inflammation associated with numerous pathological processes and the ability of each Breg cell subtype to negatively regulate immune responses as previously described. Generally, the functional mechanism of Breg cells is split into two parts; the immunomodulatory function by mediators produced by B cells and the immune effects mediated by surface molecules on B cells (see Figure 3).

### The Immunomodulatory Function of Mediator Produced by B Cells

As the hallmark of Breg cells, IL-10 cytokine is commonly used as a marker for identification of Breg cells. As an anti-inflammatory cytokine, IL-10 plays a pivotal role in controlling excessive inflammation and downregulating the immune response. Plasmacytoid dendritic cells interact with Breg cells, where they drive the differentiation of immature B cells into IL-10-producing Breg cells. Here, plasmacytoid dendritic cells releases IFN-α that interacts with CD40 on B cells. Conversely, Breg cells inhibits the production of IFN-α from plasmacytoid dendritic cells by secreting IL-10. This cross-interaction is however compromised in autoimmune diseases (31). There is a regulatory feedback loop between macrophages or microglia and Breg cells. In viral infection, B10 cells are able to inhibit microglia secreted cytokines, in addition to modulating microglial cell responses within the infected lesion (61). Moreover, B cells can dampen the activation and influence the migration of macrophages by secreting IL-10 (108). Mutually, M2 polarized microglia can enhance the proportion of Breg cells to protects against hyperactive autoimmunity (109). As for the T cells, IL-10 inhibits secretion of cytokines by Th1, thus suppressing these cells. On the other hand, IL-10 enhances polarization of Th2, thus generating and maintaining the regulatory T cell pool (57).

In addition to producing IL-10, Breg cells produce other immune-regulatory cytokines such as TGF-β and IL-35. TGF-β is a pleiotropic cytokine involved in both suppressive and inflammatory immune responses that has been shown to suppress differentiation of Th1 by inhibiting the expression of STAT4, but promotes the development of Treg cells (110). IL-35 is the newest member of the IL-12 family. It is a potent anti-inflammatory cytokine secreted by Treg cells (111). In autoimmune diseases, IL-35 can induce production of IL-10 and IL-35 by Breg cells, which inhibits pathogenic Th1/Th17 cells (112). This prevents progression of the diseases and increases the proportion of Treg cells (18). with regard to immunoregulatory property of IL-35, this cytokin, as well as its derivatives have been shown to have a therapeutic potential against autoimmune and infectious diseases (113). Besides this function, B cells are best known for their ability to produce immunoglobulins, essential in the induction of protective immunity against many pathogens. IgM promotes the removal of apoptotic cells, phagocytosis by macrophages and modulates activation of pro-inflammatory signals through the FcγR (114). Moreover, in allograft rejection, Peter I Lobo et al. found that high levels of IgM minimizes the rejection rate of renal and cardiac allografts transplants in recipient individuals. This tolerance is partially mediated by inhibition of NF-kB translocation into the nucleus by blocking TLR4. This inhibition in effect impairs differentiation of activated T cells (115). Besides IgM, IgG can suppress overwhelming immune response, important in maintaining tolerance (116). In particular, IgG4, described as “anti-inflammatory IgG” has the ability to shorten compliment processes and reduce proinflammatory responses in natural immune cells (117).

### The Immune Effects Mediated by Surface Molecules of B Cells

Immunity by cell dependent mechanism can mediate the inhibitory function of Breg cells, aided by several cell surface molecules on Breg cells. These molecules on the surface of Breg cells can induce the inhibition of immune cell function, promote differentiation of regulatory T cell and apoptosis of target cells.

CD40-activated B cells produce Foxp3^+^ Treg cells more efficiently compared with other antigen presenting cells. The longer contact time enables IL-10^+^ B cells to up-regulate expression of Foxp3 on CD4^+^ T cells, thus converts effector T cells into Treg cells. Breg cells can inhibit the proliferation of effector T cells via the CD40/CD40L interaction, suppressing autoimmune inflammation (118). Moreover, patients with the bare lymphocyte syndrome who do not express major histocompatibility complex class II (MHC II) molecules have impaired programmed cell death of autoreactive mature naive B cells, suggesting that tolerance to peripheral B cell is dependent on MHC II- TCR interaction (119). CD80 and CD86 molecules in the B7 family are expressed on antigen presenting cells and are important in establishing immune synapses and activating adaptive immune responses. Blocking antibodies against CD80 or CD86 partially reduces the regulatory effect of transitional B cells with regard to cytokine production, which implies that production of cytokines by B cells depends on interaction between B and T cells during antigen presentation (120). PD-L1 is another member of the B7 family with the ability to inhibit activation of T cell by binding to PD-1. PD-L1^hi^ Breg cells negatively regulate differentiation of T cells. Adoptive transfer of PD-L1^hi^ B cells can inhibit EAE, demonstrating that B cells can activate Treg cells through PD-L1, thus suppressing immune responses (121). In addition, Breg cells can trigger pathogenic Th1 cells to undergo apoptosis through Fas–FasL interaction and effectively downregulate pathogenic immunity of these cells (122). CD1d expressed on MZB cells are recognized by TCR on NKT cells, thus can exert their regulatory functions by activating NKT cells (123). However, in autoimmune diseases, studies have found that the expression of CD1d on B cells in individuals with SLE patients is defective. This effect impairs the presentation and recognition of glycolipids presented by CD1d via the TCRs on NKT. Consequently, this inhibits the proliferation and activation of iNKT cells. In addition, some studies have found that the proportion of iNKT cells and the expression of CD1d in B cells in individuals with SLE who positively responded to rituximab treatment were significantly increased compared with patients who did not respond to the drug.

## Bregs and Central Nervous System Inflammatory Demyelinating Diseases

### Breg Cells and Multiple Sclerosis

MS is an autoimmune disease which affects ~2.5 million people worldwide. It is characterized by chronic inflammation of CNS and aberrant infiltration of inflammatory cells that eventually leads to demyelination and axonal damage. This disease is heterogeneous and complex, and thought to be caused by interactions of genetic and environmental factors (124). The disease was originally thought to be T cell-mediated, because activated T cells were abundantly present in MS lesions. In addition, EAE could also be induced by transfer of myelin-reactive T cells. However, accumulating evidence on abnormal increase of immunoglobulin levels in the cerebrospinal fluid of affected patients, Antibody-deposition in brain lesions and the successful alleviation of the disease by B cell-depleting therapies shifted the focus to B cells as key players in immune-pathogenesis of MS. Breg cells also play a key role in demyelinating diseases of the nervous system. For instance, the occurrence of a radiologically isolated syndrome (RIS) or a CIS usually precedes MS. Further to this, individuals with RIS or CIS are more prone to develop MS within 6 months if they are deficient of IL-10-producing B cells. This suggests that Breg cells have an inhibitory effect on the progression of MS (125, 126).

The B-cell-deficient mice failed to spontaneously recover from EAE. Interestingly, this was the first study to demonstrate that B cells modulate inflammation of the immune system (5). In addition, studies have shown that Breg cells are detected in CNS of EAE in a VLA-4 dependent manner, suggesting that Breg cells may contribute to regulation of CNS autoimmunity *in situ* (127). Recently, Simon Fillatreau et al. found that hypoxia-inducible factor-1α (HIF-1α) is a critical transcription factor for the production of IL-10 by B cells, and that HIF-1α-dependent glycolysis facilitates increase in the proportion of CD1d^hi^CD5^+^ B cells. Mice with B cells lacking Hif1α have few IL-10-producing B cells, which exacerbates EAE (128).

When exploring the influence of external signals on the development and differentiation of immature B cells in the bone marrow, Simon Fillatreau et al. found that the bone marrow cells transiently stimulated by Toll-like receptor 9 can generate a new Breg cell subset CpG-proBs. CpG-proBs can slow down the development of EAE when transferred at the onset of clinical symptoms. Mechanistically, CpG-proBs can differentiate into mature Breg cells, trap T cells by releasing the CCR7 ligand and CCL19 and limit the immunopathogenesis of EAE through IL-10 production (129). Moreover, perforin-expressing regulatory B-cells (BRegs) are a new subset of Breg cells identified in patients with CIS and MS. BRegs exert their regulatory property on the disease by inhibiting proliferation of CD4^+^ T cells through the perforin/granzyme pathway (130). The proportion of BRegs in RRMS increased during relapse, suggesting that these new cells are associated with disease progression (131). Generally, the study on Breg cell subtypes and the mechanism with which they exert cell suppression have opened up interesting prospects on cell therapy during MS. Moreover, because some drugs used to treat MS patients were incidentally found to increase the production of IL-10 in human B cells, it provides promising prospect of regulating B cells for clinical treatment. As the first-line therapy against RRMS, IFN-β stimulates transformation of B lymphocytes into a subpopulation of regulatory transitional cells. Compared with untreated patients, the number of IL-10 producing transitional B lymphocytes in peripheral blood was significantly higher in IFN-β treated patients, demonstrating the role of IL-10-producing B cell populations in the disease therapy (132). Fingolimod is an immunosuppressant drug that modulates the sphingosine 1-phosphate receptor. It is the first oral, active disease-modifying drug approved for the treatment of MS. The proportion of IL-10 producing Breg cells is up-regulated while the migration capacity of the cells is enhanced after Fingolimod treatment (133). Alemtuzumab is another drug that inhibits monoclonal antibodies against CD52. It can restore the normal proportion of Breg cell subsets (CD19^+^CD24^hi^CD38^hi^ cells and CD19^+^PD-L1^hi^ cells) in the peripheral blood of patients with relapsing MS patients. This suggests that CD19^+^CD24^hi^CD38^hi^ and CD19^+^PD-L1^hi^ are promising candidate biomarkers for the efficacy of alemtuzumab therapy (134). Recently, siponimod, a selective sphingosine 1-phosphate receptor-1 and 5 modulator, was approved for active secondary progressive MS (SPMS). In one multi-centered randomized, double-blind, placebo-controlled clinical trial on SPMS patients, treatment with siponimod increased the number of transitional B cells and B1 cell subsets. In addition, the balance between Breg cells and memory B cells shifted in favor of Breg cells. Interestingly, it was a shift toward an anti-inflammatory and suppressive homeostatic immune state (135). However, in a phase 2 trial of atacicept (a recombinant fusion protein that suppresses B-cell function and proliferation), annualized relapse rates were higher in groups that received atacicept, compared to controls. One possible reason is that perhaps atacicept disrupt the B cell regulatory pathways but in turn stimulate T cell responses, a shift that sets in a proinflammatory environment and eventually relapses (136).

In conclusion, studies on the pathogenesis of MS have found that Breg cells regulates Th1/Th2 balance, induces apoptosis of effector T cells, neutralizes toxic substances, activates CD4^+^ T cells or natural killer cells (NK), inhibit the activation of dendritic cells and clear apoptotic bodies among many other functions. Further research on the Breg cell subtypes and underlying mechanism of Breg cells in MS can elucidate on the complex immune response in MS. This could provide a more comprehensive and systematic insight into its pathogenesis of the disease, and provide a basis for further exploration of new immunotherapy targets.

### Breg Cells and Neuromyelitis Optica Spectrum Disorders

Neuromyletis optica sectrum disorder (NMOSD) is a rare autoimmune disease of the CNS that primarily attacks the optic neuritis and longitudinally extended transverse myelitis (137). Epidemiologically, the disease has a globally distribution, and is more common among young and middle-aged women and results in high disability rate. A major advancement that helped to distinguish NMOSD from MS was the discovery that 75% of patients with NMOSD have detectable serum IgG auto-antibodies against the aquaporin-4 water channel (AQP4), an intergral water channel protein in astrocytes (138). After this discovery together with the pathogenic characteristics of the two diseases, it is believed that humoral mediated demyelination of astrocytes in the CNS is the major mechanism underlying the pathologenesis of the disease. The main features of the disease pathology can be reproduced using patient-derived monoclonal antibodies, thus reinforcing on the contribution of autoantibodies to the CNS injury associated with the disease (139). Moreover, studies on autoimmune diseases have found that impaired B cell tolerance potentially contributes to pathogenesis of the disease (140). In addition, accumulating evidence show that B cells play a vital role in NMOSD. This has been validated by B cell-targeted therapies such as rituximab, which have shown encouraging results for NMOSD (141).

The role of Breg cells in the pathogenesis of NMOSD has been extensively investigated. Some studies found that the proportion of Breg cells and expression of IL-10 are significantly lower in patients with NMOSD compared to those with MS, suggesting that the degree of impairment to B cell regulatory function can be considered as a distinctive marker between NMOSD and MS (142). CD19^+^CD24^hi^CD38^hi^ Breg cells are less frequent in NMOSD patients positive for AQP4 antibodies than those without these antibodies. This phenomenon is also observed in CD19^+^CD5^+^CD1d^hi^ Breg cells. In addition, NMOSD patients at acute relapse phase have lower IL-10 levels and significant impairment of CD19^+^CD24^hi^CD38^hi^ Breg cells function (143). The advent of monoclonal antibodies has also provided a new direction in the treatment of NMOSD. For example, rituximab is an anti-CD20 chimeric monoclonal antibody shown to be well-tolerated, safe and efficient, with only minor risk of mild infusion reactions among NMOSD patients (141). After rituximab treatment, the functional balance between Breg cells and memory B cells inclines toward Breg cells as opposed to pro-inflammatory cytokines producing memory B cells (17). Tocilizumab on its part is a monoclonal antibody against IL-6 receptor shown to reduce relapse rate, neuropathic pain and fatigue in patients with NMOSD (144). Patients with autoimmune diseases treated with ocilizumab show an increased expression of TGF-β and CD25 molecule on the surface of B cells, reflective of activation of Breg cells (145). In summary, decrease in the proportion of Breg cells plays a role in the pathogenesis of neuroautoimmune diseases, and the number and function of Bregs can be restored after effective treatment.

### Breg Cells and MOG-Ab Associated Demyelinating Disease

Myelin oligodendrocyte glycoprotein (MOG) is a glycoprotein located in the outer membrane of myelin, and is solely found within CNS, including in the brain, optic nerves and spinal cord. This implies that patients with encephalomyelitis attributed to MOG antibodies may develop bilateral optic nerve and lumbar spinal cord injury (146). Compared with NMOSD patients, patients with MOG antibody associated encephalomyelitis usually have a single course of the disease and show better recovery of neurological deficits after the attack. Based on clinical, immunological and histopathological evidence, encephalomyelitis associated with MOG antibodies has been regarded as a distinct disease entity different from MS and NMOSD (147).

In MOG antibody associated demyelinating disease, studies have demonstrated that the Breg cells such as CD19^+^CD24^hi^CD38^hi^ and CD19^+^CD5^+^CD1d^hi^ B cells are numerically low and functionally impaired (148). Moreover, research found that IL-6 secreted by the dendritic cells promotes the differentiation of naïve CD4 T cells into TFH cells, whereas IL-21 secreted by TFH cells induce differentiation of B cells into memory B cells and plasma cells. The latter process results in the production of antibodies and disproportion of the memory B cells/Breg cells ratio. Imbalance between the memory B cells and Breg cells promotes pro-inflammatory cytokine responses that ultimately contributes to active demyelination (148). As the first-line therapy against NMOSD, methotrexate has also been found to extend the remission period as well as reduce the recurrence rate of the disease. It also results in minor side effects. Elsewhere, methotrexate has been shown to stimulate specific immune tolerance to auto-antigens mainly by enhancing secretion of B lymphocytes that produce effector IL-10 and TGF-β (149). Studies on the role of Breg cells in the pathogenesis of MOG antibody associated demyelinating diseases remain scanty, which creates the need to explore more potential therapeutic uses of Breg cells.

## Discussion and Perspective

Regulatory B cells are important modulators of the immune response and further promotes immune tolerance. From the early immature stage to the late plasma cell stage, Breg subpopulations have been found to evolve from different stages of B cell development. Due to the different microenvironment, Breg cells have different phenotypes, but they all display immunomodulatory functions. Activation of Breg cells via BCR, TLR, or CD40 and cytokines has been shown to activate and expand the function of these cells, but mechanisms that can stabilize and maintain Breg cells remain elusive, thus there is need for further research on the plasticity and functional stability of Breg cells. In addition, numerous studies suggest that the number and function of Breg cells are involved in the pathogenesis of many diseases. Interestingly, the number and function of Breg cells among diseases and in different states are not exactly the same, which also suggests that the role of Breg cells in many disease pathologies is complicated. Therefore, it is necessary to expand our understanding on the mechanisms underlying activation, proliferation and precise functional mechanism of Breg cells in healthy individuals, as well as individuals with various immune, inflammatory or tumor diseases. Nonetheless, the important role of Breg cells in the pathogenesis of CNS IDDs has been revealed, and with the growing research on the function and contribution of Breg cells in the pathogenesis of autoimmune diseases, the therapeutic potential of Breg cells is gradually gaining acceptance. Although the therapy encompassing depletion of B-cells in treating autoimmune diseases achieved some success, this approach may exhausts Breg cells involved in the suppression of inflammation. Consequently, it would be advantageous to selectively increase Breg cells depending on the condition of the disease. In his study, Simon Fillatreau et al. suggested that reprogrammed quiescent of B cells is a novel tool for suppressing undesirable immune responses. This presents a noble research prospect for Breg cells (150). Substantially, treatment with Breg cells has certain theoretical feasibility and prospective clinical application, but its ultimate goal in the practice remains elusive.

## Consent for Publication

Written informed consent for publication was obtained from all participants.

## Author Contributions

ZQ-M and YH conceived and planned the review. ZR wrote the manuscript. ZQ-M and LY-B critically revised the manuscript for important intellectual content. All authors contributed to the article and approved the submitted version.

## Conflict of Interest

The authors declare that the research was conducted in the absence of any commercial or financial relationships that could be construed as a potential conflict of interest.

## References

[1] Hamilton JA Hsu H-C Mountz JD Autoreactive B cells in SLE, villains or innocent bystanders? Immunol Rev. (2019) 292:120–38. 10.1111/imr.12815 31631359 PMC6935412

[2] Wong FS Hu C Xiang Y Wen L. To B or not to B–pathogenic and regulatory B cells in autoimmune diabetes. Curr Opin Immunol. (2010) 22:723–31. 10.1016/j.coi.2010.10.002 21050736

[3] Fillatreau S. Pathogenic functions of B cells in autoimmune diseases: IFN-γ production joins the criminal gang. Eur J Immunol. (2015) 45:966–70. 10.1002/eji.201545544 25727209

[4] Katz SI Parker D Turk JL. B-cell suppression of delayed hypersensitivity reactions. Nature. (1974) 251:550–1. 10.1038/251550a0 4547522

[5] Wolf SD Dittel BN Hardardottir F Janeway CAJ. Experimental autoimmune encephalomyelitis induction in genetically B cell-deficient mice. J Exp Med. (1996) 184:2271–8. 10.1084/jem.184.6.2271 8976182 PMC2196394

[6] Mizoguchi A Mizoguchi E Takedatsu H Blumberg RS Bhan AK. Chronic intestinal inflammatory condition generates IL-10-producing regulatory B cell subset characterized by CD1d upregulation. Immunity. (2002) 16:219–30. 10.1016/S1074-7613(02)00274-1 11869683

[7] Mauri C Gray D Mushtaq N Londei M. Prevention of arthritis by interleukin 10-producing B cells. J Exp Med. (2003) 197:489–501. 10.1084/jem.20021293 12591906 PMC2193864

[8] Bettelli E Das MP Howard E Weiner H Sobel R Kuchroo V. IL-10 is critical in the regulation of automimmune encephalomyelitis as demonstrated by studies of IL-10 and IL-4 deficient and transgenic mice. J Neuroimmunol. (1998) 90:54. 10.1016/S0165-5728(98)91500-4 9759845

[9] Samoilova EB Horton JL Chen Y. Acceleration of experimental autoimmune encephalomyelitis in interleukin-10-deficient mice: roles of interleukin-10 in disease progression and recovery. Cell Immunol. (1998) 188:118–24. 10.1006/cimm.1998.1365 9756642

[10] Fillatreau S Sweenie CH McGeachy MJ Gray D Anderton SM. B cells regulate autoimmunity by provision of IL-10. Nat Immunol. (2002) 3:944–50. 10.1038/ni833 12244307

[11] Yanaba K Bouaziz J-DD Haas KM Poe JC Fujimoto M Tedder TF. A regulatory B cell subset with a unique CD1dhiCD5+ phenotype controls T cell-dependent inflammatory responses. Immunity. (2008) 28:639–50. 10.1016/j.immuni.2008.03.017 18482568

[12] Ray A Wang L Dittel BN. IL-10-independent regulatory B-cell subsets and mechanisms of action. Int Immunol. (2015) 27:531–6. 10.1093/intimm/dxv033 25999596 PMC11513724

[13] Reindl M Waters P Myelin oligodendrocyte glycoprotein antibodies in neurological disease. Nat Rev Neurol. (2019) 15:89–102. 10.1038/s41582-018-0112-x 30559466

[14] Hu Y Nie H Yu H-H Qin C Wu L-J Tang Z. Efficacy and safety of rituximab for relapsing-remitting multiple sclerosis: a systematic review and meta-analysis. Autoimmun Rev. (2019) 18:542–8. 10.1016/j.autrev.2019.03.011 30844555

[15] Status ED Damato V Evoli A Iorio R Efficacy and safety of rituximab therapy in neuromyelitis optica spectrum disorders: a systematic review and meta-analysis. JAMA Neurol. (2016) 73:1342–8. 10.1001/jamaneurol.2016.1637 27668357

[16] Thi Cuc B Pohar J Fillatreau S. Understanding regulatory B cells in autoimmune diseases: the case of multiple sclerosis. Curr Opin Immunol. (2019) 61:26–32. 10.1016/j.coi.2019.07.007 31445312

[17] Quan C ZhangBao J Lu J Zhao C Cai T Wang B The immune balance between memory and regulatory B cells in NMO and the changes of the balance after methylprednisolone or rituximab therapy. J Neuroimmunol. (2015) 282:45–53. 10.1016/j.jneuroim.2015.03.016 25903728

[18] Wang R-X Yu C-R Dambuza IM Mahdi RM Dolinska MB Sergeev YV. Interleukin-35 induces regulatory B cells that suppress autoimmune disease. Nat Med. (2014) 20:633–41. 10.1038/nm.3554 24743305 PMC4048323

[19] Dambuza IM He C Choi JK Yu C-RR Wang R Mattapallil MJ. IL-12p35 induces expansion of IL-10 and IL-35-expressing regulatory B cells and ameliorates autoimmune disease. Nat Commun. (2017) 8:719. 10.1038/s41467-017-00838-4 28959012 PMC5620058

[20] Xue J-M Ma F An Y-F Suo L-M Geng X-R Song Y-N. Probiotic extracts ameliorate nasal allergy by inducing interleukin-35-producing dendritic cells in mice. Int Forum Allergy Rhinol. (2019) 9:1289–96. 10.1002/alr.22438 31623025

[21] Hagn M Ebel V Sontheimer K Schwesinger E Lunov O Beyer T. CD5+ B cells from individuals with systemic lupus erythematosus express granzyme B. Eur J Immunol. (2010) 40:2060–9. 10.1002/eji.200940113 20394077

[22] Yoshizaki A Miyagaki T DiLillo DJ Matsushita T Horikawa M Kountikov EI. Regulatory B cells control T-cell autoimmunity through IL-21-dependent cognate interactions. Nature. (2012) 491:264–8. 10.1038/nature11501 23064231 PMC3493692

[23] Rosser EC Oleinika K Tonon S Doyle R Bosma A Carter NA. Regulatory B cells are induced by gut microbiota-driven interleukin-1beta and interleukin-6 production. Nat Med. (2014) 20:1334–9. 10.1038/nm.3680 25326801

[24] Rafei M Hsieh J Zehntner S Li M Forner K Birman E. A granulocyte-macrophage colony-stimulating factor and interleukin-15 fusokine induces a regulatory B cell population with immune suppressive properties. Nat Med. (2009) 15:1038–45. 10.1038/nm.2003 19668193

[25] Haeberlein S Obieglo K Ozir-Fazalalikhan A Chayé MAM Veninga H van der Vlugt. Schistosome egg antigens, including the glycoprotein IPSE/alpha-1, trigger the development of regulatory B cells. PLoS Pathog. (2017) 13:e1006539. 10.1371/journal.ppat.1006539 28753651 PMC5550006

[26] Ristori G Romano S Cannoni S Visconti A Tinelli E Mendozzi L. Effects of bacille calmette-guerin after the first demyelinating event in the CNS. Neurology. (2014) 82:41–8. 10.1212/01.wnl.0000438216.93319.ab 24306002 PMC3873620

[27] Paolillo A Buzzi MG Giugni E Sabatini U Bastianello S Pozzilli C. The effect of bacille calmette-guerin on the evolution of new enhancing lesions to hypointense T1 lesions in relapsing remitting MS. J Neurol. (2003) 250:247–8. 10.1007/s00415-003-0967-6 12622098

[28] Gray M Miles K Salter D Gray D Savill J. Apoptotic cells protect mice from autoimmune inflammation by the induction of regulatory B cells. Proc Natl Acad Sci USA. (2007) 104:14080–5. 10.1073/pnas.0700326104 17715067 PMC1955797

[29] Miles K Heaney J Sibinska Z Salter D Savill J Gray D. A tolerogenic role for toll-like receptor 9 is revealed by B-cell interaction with DNA complexes expressed on apoptotic cells. Proc Natl Acad Sci USA. (2012) 109:887–92. 10.1073/pnas.1109173109 22207622 PMC3271931

[30] Komlósi ZI Kovács N van de Veen W Kirsch AI Fahrner HB Wawrzyniak M. Human CD40 ligand–expressing type 3 innate lymphoid cells induce IL-10–producing immature transitional regulatory B cells. J Allergy Clin Immunol. (2018) 142:178–94.e11. 10.1016/j.jaci.2017.07.046 28939410

[31] Menon M Blair PA Isenberg DA Mauri C Menon M Blair PA Article A regulatory feedback between plasmacytoid dendritic cells and regulatory B cells is aberrant in systemic lupus erythematosus article A regulatory feedback between plasmacytoid dendritic cells and regulatory B cells is aberrant in systemic lupus E. Immunity. (2016) 44:683–97. 10.1016/j.immuni.2016.02.012 26968426 PMC4803914

[32] Kuan Y-C Wu Y-J Hung C-L Sheu F. Trametes versicolor protein YZP activates regulatory B lymphocytes - gene identification through de novo assembly and function analysis in a murine acute colitis model. PLoS ONE. (2013) 8:e72422. 10.1371/journal.pone.0072422 24019869 PMC3760908

[33] Liu X Huang H Gao H Wu X Zhang W Yu B. Regulatory B cells induced by ultraviolet B through toll-like receptor 4 signalling contribute to the suppression of contact hypersensitivity responses in mice. Contact Dermatitis. (2018) 78:117–30. 10.1111/cod.12913 29205369

[34] Wang T Marken J Chen J Tran VB Li Q-Z Li M. High TLR7 expression drives the expansion of CD19^+^CD24^hi^CD38^hi^ transitional B cells and autoantibody production in SLE patients. Front Immunol. (2019) 10:1243. 10.3389/fimmu.2019.01243 31231380 PMC6559307

[35] Liu BS Cao Y Huizinga TW Hafler DA Toes REM. TLR-mediated STAT3 and ERK activation controls IL-10 secretion by human B cells. Eur J Immunol. (2014) 44:2121–9. 10.1002/eji.201344341 24737107

[36] Neves P Lampropoulou V Calderon-Gomez E Roch T Stervbo U Shen P. Signaling via the MyD88 adaptor protein in B cells suppresses protective immunity during salmonella typhimurium infection. Immunity. (2010) 33:777–90. 10.1016/j.immuni.2010.10.016 21093317

[37] Sayi A Kohler E Toller IM Flavell RA Müller W Roers A. TLR-2–activated B cells suppress helicobacter -induced preneoplastic gastric immunopathology by inducing T regulatory-1 cells. J Immunol. (2011) 186:878–90. 10.4049/jimmunol.1002269 21149607

[38] Cohen-Sfady M Nussbaum G Pevsner-Fischer M Mor F Carmi P Zanin-Zhorov A. Heat shock protein 60 activates B cells via the TLR4-MyD88 pathway. J Immunol. (2005) 175:3594–602. 10.4049/jimmunol.175.6.3594 16148103

[39] Hong J Fang J Lan R Tan Q Tian Y Zhang M. TLR9 mediated regulatory B10 cell amplification following sub-total body irradiation: implications in attenuating EAE. Mol Immunol. (2017) 83:52–61. 10.1016/j.molimm.2017.01.011 28110075

[40] Oka A Mishima Y Liu B Herzog JW Steinbach EC Kobayashi T. Phosphoinositide 3-kinase P110δ-signaling is critical for microbiota-activated IL-10 production by B cells that regulate intestinal inflammation. Cells. (2019) 8:1121. 10.3390/cells8101121 31546615 PMC6829312

[41] Zhou M Wen Z Cheng F Ma J Li W Ren H. Tumor-released autophagosomes induce IL-10-producing B cells with suppressive activity on T lymphocytes via TLR2-MyD88-NF-kappaB signal pathway. Oncoimmunology. (2016) 5:e1180485. 10.1080/2162402X.2016.1180485 27622036 PMC5006924

[42] Kuwata H Matsumoto M Atarashi K Morishita H Hirotani T Koga R. IkappaBNS inhibits induction of a subset of Toll-like receptor-dependent genes and limits inflammation. Immunity. (2006) 24:41–51. 10.1016/j.immuni.2005.11.004 16413922

[43] Miura M Hasegawa N Noguchi M Sugimoto K Touma M. The atypical IκB protein IκBNS is important for toll-like receptor-induced interleukin-10 production in B cells. Immunology. (2016) 147:453–63. 10.1111/imm.12578 26749055 PMC4799890

[44] Blair PA Norena LY Flores-Borja F Rawlings DJ Isenberg DA Ehrenstein MR. CD19^+^CD24^hi^CD38^hi^ B cells exhibit regulatory capacity in healthy individuals but are functionally impaired in systemic lupus erythematosus patients. Immunity. (2010) 32:129–40. 10.1016/j.immuni.2009.11.009 20079667

[45] Blair PA Chavez-Rueda KA Evans JG Shlomchik MJ Eddaoudi A Isenberg DA. Selective targeting of B cells with agonistic anti-CD40 is an efficacious strategy for the generation of induced regulatory T2-like B cells and for the suppression of lupus in MRL/ lpr mice. J Immunol. (2009) 182:3492–502. 10.4049/jimmunol.0803052 19265127 PMC4082659

[46] Okada Y Ochi H Fujii C Hashi Y Hamatani M Ashida S. Signaling via toll-like receptor 4 and CD40 in B cells plays a regulatory role in the pathogenesis of multiple sclerosis through interleukin-10 production. J Autoimmun. (2018) 88:103–13. 10.1016/j.jaut.2017.10.011 29146546

[47] Tang Y Jiang Q Ou Y Zhang F Qing K Sun Y. BIP induces mice CD19^hi^ regulatory B cells producing IL-10 and highly expressing PD-L1, FasL. Mol Immunol. (2016) 69:44–51. 10.1016/j.molimm.2015.10.017 26655428

[48] Hussain S Delovitch TL. Intravenous transfusion of BCR-activated B cells protects NOD mice from type 1 diabetes in an IL-10-dependent manner. J Immunol. (2007) 179:7225–32. 10.4049/jimmunol.179.11.7225 18025164

[49] Matsumoto M Fujii Y Baba A Hikida M Kurosaki T Baba Y. The calcium sensors STIM1 and STIM2 control B cell regulatory function through interleukin-10 production. Immunity. (2011) 34:703–14. 10.1016/j.immuni.2011.03.016 21530328

[50] Matsumoto M Baba A Yokota T Nishikawa H Ohkawa Y Kayama H. Interleukin-10-producing plasmablasts exert regulatory function in autoimmune inflammation. Immunity. (2014) 41:1040–51. 10.1016/j.immuni.2014.10.016 25484301

[51] Burlock B Richardson G Garcia-Rodriguez S Guerrero S Zubiaur M Sancho J. The role of CD38 on the function of regulatory B cells in a murine model of lupus. Int J Mol Sci. (2018) 19:2906. 10.3390/ijms19102906 30257456 PMC6213330

[52] Domínguez-Pantoja M López-Herrera G Romero-Ramírez H Santos-Argumedo L Chávez-Rueda AK Hernández-Cueto. CD38 protein deficiency induces autoimmune characteristics and its activation enhances IL-10 production by regulatory B cells. Scand J Immunol. (2018) 87:1–11. 10.1111/sji.12664 29603313

[53] Alhabbab R Blair P Smyth LA Ratnasothy K Peng Q Moreau A. Galectin-1 is required for the regulatory function of B cells. Sci Rep. (2018) 8:2725. 10.1038/s41598-018-19965-z 29426942 PMC5807431

[54] Moreau A Blair PA Chai J-G Ratnasothy K Stolarczyk E Alhabbab R. Transitional-2 B cells acquire regulatory function during tolerance induction and contribute to allograft survival. Eur J Immunol. (2015) 45:843–53. 10.1002/eji.201445082 25408265

[55] Bankoti R Gupta K Levchenko A Stäger S Stager S. Marginal zone B cells regulate antigen-specific T cell responses during infection. J Immunol. (2012) 188:3961–71. 10.4049/jimmunol.1102880 22412197

[56] Evans JG Chavez-Rueda KA Eddaoudi A Meyer-Bahlburg A Rawlings DJ Ehrenstein MR. Novel suppressive function of transitional 2 B cells in experimental arthritis. J Immunol. (2007) 178:7868–78. 10.4049/jimmunol.178.12.7868 17548625

[57] Carter NA Vasconcellos R Rosser EC Tulone C Munoz-Suano A Kamanaka M. Mice lacking endogenous IL-10-producing regulatory B cells develop exacerbated disease and present with an increased frequency of Th1/Th17 but a decrease in regulatory T cells. J Immunol. (2011) 186:5569–79. 10.4049/jimmunol.1100284 21464089

[58] Ganti SN Albershardt TC Iritani BM Ruddell A. Regulatory B cells preferentially accumulate in tumor-draining lymph nodes and promote tumor growth. Sci Rep. (2015) 5:12255. 10.1038/srep12255 26193241 PMC4507466

[59] Yang M Deng J Liu Y Ko K-H Wang X Jiao Z. IL-10-producing regulatory B10 cells ameliorate collagen-induced arthritis via suppressing Th17 cell generation. Am J Pathol. (2012) 180:2375–85. 10.1016/j.ajpath.2012.03.010 22538089

[60] Chen Y Li C Lu Y Zhuang H Gu W Liu B. IL-10-Producing CD1d^hi^CD5^+^ regulatory B cells may play a critical role in modulating immune homeostasis in silicosis patients. Front Immunol. (2017) 8:110. 10.3389/fimmu.2017.00110 28243231 PMC5303715

[61] Mutnal MB Hu S Schachtele SJ Lokensgard JR. Infiltrating regulatory B cells control neuroinflammation following viral brain infection. J Immunol. (2014) 193:6070–80. 10.4049/jimmunol.1400654 25385825 PMC4258482

[62] Das S Bar-Sagi D. BTK signaling drives CD1d^hi^CD5^+^ regulatory B-cell differentiation to promote pancreatic carcinogenesis. Oncogene. (2019) 38:3316–24. 10.1038/s41388-018-0668-3 30635655 PMC6486434

[63] Miles K Simpson J Brown S Cowan G Gray D Gray M. Immune tolerance to apoptotic self is mediated primarily by regulatory B1a cells. Front Immunol. (2018) 8:1952. 10.3389/fimmu.2017.01952 29403471 PMC5780629

[64] Maseda D Candando KM Smith SH Kalampokis I Weaver CT Plevy SE. Peritoneal cavity regulatory B cells (B10 cells) modulate IFN-γ+CD4+ T cell numbers during colitis development in mice. J Immunol. (2013) 191:2780–95. 10.4049/jimmunol.1300649 23918988 PMC3770313

[65] Lundy SK Fox DA. Reduced fas ligand-expressing splenic CD5+ B lymphocytes in severe collagen-induced arthritis. Arthritis Res Ther. (2009) 11:R128. 10.1186/ar2795 19706160 PMC2745812

[66] Kobayashi T Oishi K Okamura A Maeda S Komuro A Hamaguchi Y. Regulatory B1a cells suppress melanoma tumor immunity via IL-10 production and inhibiting T helper type 1 cytokine production in tumor-infiltrating CD8^+^ T cells. J Invest Dermatol. (2019) 139:1535–44.e1. 10.1016/j.jid.2019.02.016 30836062

[67] Shen P Roch T Lampropoulou V Connor RAO Stervbo U Hilgenberg E. IL-35-producing B cells are critical regulators of immunity during autoimmune and infectious diseases. Nature. (2014) 507:366–70. 10.1038/nature12979 24572363 PMC4260166

[68] Wei Y Lao X-M Xiao X Wang X-Y Wu Z-J Zeng Q-H. Plasma cell polarization to the immunoglobulin G phenotype in hepatocellular carcinomas involves epigenetic alterations and promotes hepatoma progression in mice. Gastroenterology. (2019) 156:1890–904.e16. 10.1053/j.gastro.2019.01.250 30711627

[69] Zacca ER Onofrio LI Acosta CDV Ferrero PV Alonso SM Ramello MC. PD-L1^+^ regulatory B cells are significantly decreased in rheumatoid arthritis patients and increase after successful treatment. Front Immunol. (2018) 9:2241. 10.3389/fimmu.2018.02241 30327652 PMC6174216

[70] Ding Q Yeung M Camirand G Zeng Q Akiba H Yagita H. Regulatory B cells are identified by expression of TIM-1 and can be induced through TIM-1 ligation to promote tolerance in mice. J Clin Invest. (2011) 121:3645–56. 10.1172/JCI46274 21821911 PMC3163958

[71] Xiao S Brooks CR Sobel RA Kuchroo VK. Tim-1 is essential for induction and maintenance of IL-10 in regulatory B cells and their regulation of tissue inflammation. J Immunol. (2015) 194:1602–8. 10.4049/jimmunol.1402632 25582854 PMC4346345

[72] Saze Z Schuler PJ Hong C-S Cheng D Jackson EK Whiteside TL. Adenosine production by human B cells and B cell-mediated suppression of activated T cells. Blood. (2013) 122:9–18. 10.1182/blood-2013-02-482406 23678003 PMC3701906

[73] Khoder A Sarvaria A Alsuliman A Chew C Sekine T Cooper N. Regulatory B cells are enriched within the IgM memory and transitional subsets in healthy donors but are deficient in chronic GVHD. Blood. (2014) 124:2034–45. 10.1182/blood-2014-04-571125 25051962 PMC4186534

[74] Hua C Audo R Yeremenko N Baeten D Hahne M Combe B. A proliferation inducing ligand (APRIL) promotes IL-10 production and regulatory functions of human B cells. J Autoimmun. (2016) 73:64–72. 10.1016/j.jaut.2016.06.002 27372914

[75] Salomon S Guignant C Morel P Flahaut G Brault C Gourguechon C. Th17 and CD24^hi^CD27^+^ regulatory B lymphocytes are biomarkers of response to biologics in rheumatoid arthritis. Arthritis Res Ther. (2017) 19:33. 10.1186/s13075-017-1244-x 28183330 PMC5301325

[76] Rabani M Wilde B Hübbers K Xu S Kribben A Witzke O. IL-21 dependent granzyme B production of B-cells is decreased in patients with lupus nephritis. Clin Immunol. (2018) 188:45–51. 10.1016/j.clim.2017.12.005 29274388

[77] Kaltenmeier C Gawanbacht A Beyer T Lindner S Trzaska T van der Merwe JA. CD4+ T cell-derived IL-21 and deprivation of CD40 signaling favor the *in vivo* development of granzyme B-expressing regulatory B cells in HIV patients. J Immunol. (2015) 194:3768–77. 10.4049/jimmunol.1402568 25780036

[78] Lindner S Dahlke K Sontheimer K Hagn M Kaltenmeier C Barth TFEE. Interleukin 21-induced granzyme b-expressing b cells infiltrate tumors and regulate t cells. Cancer Res. (2013) 73:2468–79. 10.1158/0008-5472.CAN-12-3450 23384943

[79] Kim AS Doherty TA Karta MR Das S Baum R Rosenthal P. Regulatory B cells and T follicular helper cells are reduced in allergic rhinitis. J Allergy Clin Immunol. (2016) 138:1192–5.e5. 10.1016/j.jaci.2016.03.017 27142393 PMC5053844

[80] Kubo S Yamada T Osawa Y Ito Y Narita N Fujieda S. Cytosine-phosphate-guanosine-DNA induces CD274 expression in human B cells and suppresses T helper type 2 cytokine production in pollen antigen-stimulated CD4-positive cells. Clin Exp Immunol. (2012) 169:1–9. 10.1111/j.1365-2249.2012.04585.x 22670772 PMC3390467

[81] Liu R Lu Z Gu J Liu J Huang E Liu X. MicroRNAs 15A and 16-1 activate signaling pathways that mediate chemotaxis of immune regulatory B cells to colorectal tumors. Gastroenterology. (2018) 154:637–51.e7. 10.1053/j.gastro.2017.09.045 29031499

[82] Colgan SP Hershberg RM Furuta GT Blumberg RS. Ligation of intestinal epithelial CD1d induces bioactive IL-10: critical role of the cytoplasmic tail in autocrine signaling. Proc Natl Acad Sci USA. (1999) 96:13938–43. 10.1073/pnas.96.24.13938 10570177 PMC24169

[83] Kovesdi D Paszty K Enyedi A Kiss E Matko J Ludanyi K. Antigen receptor-mediated signaling pathways in transitional immature B cells. Cell Signal. (2004) 16:881–9. 10.1016/j.cellsig.2004.01.005 15157667

[84] Yang M Sun L Wang S Ko K-H Xu H Zheng B-J. Novel function of B cell-activating factor in the induction of IL-10-producing regulatory B cells. J Immunol. (2010) 184:3321–5. 10.4049/jimmunol.0902551 20208006

[85] Huber K Sármay G Kövesdi D. MZ B cells migrate in a T-bet dependent manner and might contribute to the remission of collagen-induced arthritis by the secretion of IL-10. Eur J Immunol. (2016) 46:2239–46. 10.1002/eji.201546248 27343199

[86] Hsu LH Li KP Chu KH Chiang BL. A B-1a cell subset induces Foxp3-T cells with regulatory activity through an IL-10-independent pathway. Cell Mol Immunol. (2015) 12:354–65. 10.1038/cmi.2014.56 25132452 PMC4654317

[87] Rojas OL Pröbstel A-K Porfilio EA Wang AA Charabati M Sun T. Recirculating intestinal IgA-producing cells regulate neuroinflammation via IL-10. Cell. (2019) 176:610–24.e18. 10.1016/j.cell.2018.11.035 30612739 PMC6903689

[88] Suzuki-Yamazaki N Yanobu-Takanashi R Okamura T Takaki S. IL-10 production in murine IgM^+^ CD138^hi^ cells is driven by Blimp-1 and downregulated in class-switched cells. Eur J Immunol. (2017) 47:493–503. 10.1002/eji.201646549 28012163

[89] Lino AC Dang VD Lampropoulou V Welle A Joedicke J Pohar J. LAG-3 inhibitory receptor expression identifies immunosuppressive natural regulatory plasma cells. Immunity. (2018) 49:120–33.e9. 10.1016/j.immuni.2018.06.007 30005826 PMC6057275

[90] Aravena O Ferrier A Menon M Mauri C Aguillon JC Soto L. TIM-1 defines a human regulatory B cell population that is altered in frequency and function in systemic sclerosis patients. Arthritis Res Ther. (2017) 19:8. 10.1186/s13075-016-1213-9 28103916 PMC5248463

[91] Hu Y Yu P Yu X Hu X Kawai T Han X. IL-21/anti-Tim1/CD40 ligand promotes B10 activity *in vitro* and alleviates bone loss in experimental periodontitis *in vivo*. Biochim Biophys Acta Mol Basis Dis. (2017) 1863:2149–57. 10.1016/j.bbadis.2017.06.001 28583714 PMC5567687

[92] Kaku H Cheng KF Al-Abed Y Rothstein TL. A novel mechanism of B cell-mediated immune suppression through CD73 expression and adenosine production. J Immunol. (2014) 193:5904–13. 10.4049/jimmunol.1400336 25392527 PMC4321875

[93] Lin X Wang X Xiao F Ma K Liu L Wang X. IL-10-producing regulatory B cells restrain the T follicular helper cell response in primary Sjögren's syndrome. Cell Mol Immunol. (2019) 16:921–31. 10.1038/s41423-019-0227-z 30948793 PMC6884445

[94] Li T Yu Z Qu Z Zhang N Crew R Jiang Y. Decreased number of CD19^+^CD24^hi^CD38^hi^ regulatory B cells in diabetic nephropathy. Mol Immunol. (2019) 112:233–9. 10.1016/j.molimm.2019.05.014 31181422

[95] Mauri C Bosma A. Immune regulatory function of B cells. Annu Rev Immunol. (2012) 30:221–41. 10.1146/annurev-immunol-020711-074934 22224776

[96] Newell KA Asare A Kirk AD Gisler TD Bourcier K Suthanthiran M. Identification of a B cell signature associated with renal transplant tolerance in humans. J Clin Invest. (2010) 120:1836–47. 10.1172/JCI39933 20501946 PMC2877933

[97] Siewe B Stapleton JT Martinson J Keshavarzian A Kazmi N Demarais PM. Regulatory B cell frequency correlates with markers of HIV disease progression and attenuates anti-HIV CD8^+^ T cell function *in vitro*. J Leukoc Biol. (2013) 93:811–18. 10.1189/jlb.0912436 23434518 PMC3629440

[98] Hasan MM Thompson-Snipes L Klintmalm G Demetris AJ O'Leary J Oh S. CD24^hi^CD38^hi^ and CD24^hi^CD27^+^ human regulatory B cells display common and distinct functional characteristics. J Immunol. (2019) 203:2110–20. 10.4049/jimmunol.1900488 31511354

[99] Zheng Y Ge W Ma Y Xie G Wang W Han L. miR-155 regulates IL-10-producing CD24^hi^CD27^+^ B cells and impairs their function in patients with Crohn's disease. Front Immunol. (2017) 8:914. 10.3389/fimmu.2017.00914 28824639 PMC5540954

[100] Liu Z Dang E Li B Qiao H Jin L Zhang J. Dysfunction of CD19^+^CD24^hi^CD27^+^ B regulatory cells in patients with bullous pemphigoid. Sci Rep. (2018) 8:703. 10.1038/s41598-018-19226-z 29335495 PMC5768798

[101] De Masson A Bouaziz J-DD Le Buanec H Robin M O'Meara A Parquet N. CD24^hi^CD27^+^ and plasmablast-like regulatory B cells in human chronic graft-versus-host disease. Blood. (2015) 125:1830–39. 10.1182/blood-2014-09-599159 25605369

[102] Zhou H Zhan F Zhang H Gu J Mu X Gao J. The proportion of CD19+CD24hiCD27+ regulatory B cells predicts the occurrence of acute allograft rejection in liver transplantation. Ann Transl Med. (2019) 7:465. 10.21037/atm.2019.08.05 31700901 PMC6803179

[103] Hagn M Jahrsdörfer B. Why do human B cells secrete granzyme B? Insights into a novel B-cell differentiation pathway. Oncoimmunology. (2012) 1:1368–75. 10.4161/onci.22354 23243600 PMC3518509

[104] Xu L Liu X Liu H Zhu L Zhu H Zhang J. Impairment of granzyme B-producing regulatory B cells correlates with exacerbated rheumatoid arthritis. Front Immunol. (2017) 8:768. 10.3389/fimmu.2017.00768 28713386 PMC5491972

[105] Zhu J Zeng Y Dolff S Bienholz A Lindemann M Brinkhoff A. Granzyme B producing B-cells in renal transplant patients. Clin Immunol. (2017) 184:48–53. 10.1016/j.clim.2017.04.016 28461110

[106] van de Veen W Stanic B Wirz OF Jansen K Globinska A Akdis MM. Role of regulatory B cells in immune tolerance to allergens and beyond. J Allergy Clin Immunol. (2016) 138:654–65. 10.1016/j.jaci.2016.07.006 27596706

[107] van de Veen W Stanic B Yaman G Wawrzyniak M Söllner S Akdis DG. IgG4 production is confined to human IL-10-producing regulatory B cells that suppress antigen-specific immune responses. J Allergy Clin Immunol. (2013) 131:1204–12. 10.1016/j.jaci.2013.01.014 23453135

[108] Pennati A Asress S Glass JD Galipeau J. Adoptive transfer of IL-10^+^ regulatory B cells decreases myeloid-derived macrophages in the central nervous system in a transgenic amyotrophic lateral sclerosis model. Cell Mol Immunol. (2018) 15:727–30. 10.1038/cmi.2017.152 29307886 PMC6123431

[109] Benedek G Zhang J Nguyen H Kent G Seifert H Vandenbark AA. Novel feedback loop between M2 macrophages/microglia and regulatory B cells in estrogen-protected EAE mice. J Neuroimmunol. (2017) 305:59–67. 10.1016/j.jneuroim.2016.12.018 28284347 PMC5387865

[110] Schmitt N Liu Y Bentebibel S-E Munagala I Bourdery L Venuprasad K. The cytokine TGF-β co-opts signaling via STAT3-STAT4 to promote the differentiation of human TFH cells. Nat Immunol. (2014) 15:856–65. 10.1038/ni.2947 25064073 PMC4183221

[111] Collison LW Vignali DAA Interleukin-35: odd one out or part of the family? Immunol Rev. (2008) 226:248–62. 10.1111/j.1600-065X.2008.00704.x 19161429 PMC2631363

[112] Mauri C Nistala K. Interleukin-35 takes the “B” line. Nat Med. (2014) 20:580–81. 10.1038/nm.3594 24901562

[113] Kouzaki H Arikata M Koji M Arai H Yamamoto S Kikuoka H. Dynamic change of anti-inflammatory cytokine IL-35 in allergen immune therapy for Japanese cedar pollinosis. Allergy. (2019) 75:981–83. 10.1111/all.14124 31755994

[114] Litvack ML Post M Palaniyar N. IgM promotes the clearance of small particles and apoptotic microparticles by macrophages. PLoS ONE. (2011) 6:e17223. 10.1371/journal.pone.0017223 21448268 PMC3063157

[115] Lobo PI Bajwa A Schlegel KH Vengal J Lee SJ Huang L. Natural IgM anti-leukocyte autoantibodies attenuate excess inflammation mediated by innate and adaptive immune mechanisms involving Th-17. J Immunol. (2012) 188:1675–85. 10.4049/jimmunol.1101762 22262657 PMC3273570

[116] Bolland S. A newly discovered Fc receptor that explains IgG-isotype disparities in effector responses. Immunity. (2005) 23:2–4. 10.1016/j.immuni.2005.07.002 16039573

[117] Rincón-Arévalo H Sanchez-Parra CC Castanõ D Yassin L Vásquez G Casta D. Regulatory B cells and mechanisms. Int Rev Immunol. (2016) 35:156–76. 10.3109/08830185.2015.1015719 25793964

[118] Lemoine S Morva A Youinou P Jamin C. Human T cells induce their own regulation through activation of B cells. J Autoimmun. (2011) 36:228–38. 10.1016/j.jaut.2011.01.005 21316922

[119] Hervé M Isnardi I Ng Y Bussel JB Ochs HD Cunningham-Rundles C. CD40 ligand and MHC class II expression are essential for human peripheral B cell tolerance. J Exp Med. (2007) 204:1583–93. 10.1084/jem.20062287 17562816 PMC2118633

[120] Flores-Borja F Bosma A Ng D Reddy V Ehrenstein MR Isenberg DA. CD19+CD24hiCD38hi B cells maintain regulatory T cells while limiting TH1 and TH17 differentiation. Sci Transl Med. (2013) 5:173ra23. 10.3410/f.717980821.793472337 23427243

[121] Khan AR Hams E Floudas A Sparwasser T Weaver CT Fallon PG. PD-L1hi B cells are critical regulators of humoral immunity. Nat Commun. (2015) 6:5997. 10.1038/ncomms6997 25609381

[122] Mann MK Maresz K Shriver LP Tan Y Dittel BN. B cell regulation of CD4 + CD25 + T regulatory cells and IL-10 via B7 is essential for recovery from experimental autoimmune encephalomyelitis. J Immunol. (2007) 178:3447–56. 10.4049/jimmunol.178.6.3447 17339439

[123] Bosma A Abdel-Gadir A Isenberg DA Jury EC Mauri C. Lipid-antigen presentation by CD1d^+^ B cells is essential for the maintenance of invariant natural killer T cells. Immunity. (2012) 36:477–90. 10.1016/j.immuni.2012.02.008 22406267 PMC3391684

[124] Milo R Miller A. Revised diagnostic criteria of multiple sclerosis. Autoimmun Rev. (2014) 13:518–24. 10.1016/j.autrev.2014.01.012 24424194

[125] Guerrier T Labalette M Launay D Lee-Chang C Outteryck O Lefèvre G. Proinflammatory B-cell profile in the early phases of MS predicts an active disease. Neurol Neuroimmunol neuroinflammation. (2017) 5:e431. 10.1212/NXI.0000000000000431 29296635 PMC5745361

[126] Barun B Bar-Or A. Treatment of multiple sclerosis with anti-CD20 antibodies. Clin Immunol. (2012) 142:31–7. 10.1016/j.clim.2011.04.005 21555250

[127] Lehmann-Horn K Sagan SA Winger RC Spencer CM Bernard CCA Sobel RA. CNS accumulation of regulatory B cells is VLA-4-dependent. Neurol Neuroimmunol NeuroInflammation. (2016) 3:e212. 10.1212/NXI.0000000000000212 27027096 PMC4794810

[128] Meng X Grotsch B Luo Y Knaup KX Wiesener MS Chen X-X. Hypoxia-inducible factor-1alpha is a critical transcription factor for IL-10-producing B cells in autoimmune disease. Nat Commun. (2018) 9:251. 10.1038/s41467-017-02683-x 29343683 PMC5772476

[129] Korniotis S Gras C Letscher H Montandon R Megret J Siegert S. Treatment of ongoing autoimmune encephalomyelitis with activated B-cell progenitors maturing into regulatory B cells. Nat Commun. (2016) 7:12134. 10.1038/ncomms12134 27396388 PMC4942579

[130] de Andrés C Tejera-Alhambra M Alonso BB Valor L Teijeiro R Ramos-Medina RR. New regulatory CD19^+^CD25^+^ B-cell subset in clinically isolated syndrome and multiple sclerosis relapse. Changes after glucocorticoids. J Neuroimmunol. (2014) 270:37–44. 10.1016/j.jneuroim.2014.02.003 24662004

[131] Montandon R Korniotis S Layseca-Espinosa E Gras C Mégret J Ezine S. Innate pro-B-cell progenitors protect against type 1 diabetes by regulating autoimmune effector T cells. Proc Natl Acad Sci USA. (2013) 110:E2199–208. 10.1073/pnas.1222446110 23716674 PMC3683765

[132] Schubert RD Hu Y Kumar G Szeto S Abraham P Winderl J. IFN-beta treatment requires B cells for efficacy in neuroautoimmunity. J Immunol. (2015) 194:2110–16. 10.4049/jimmunol.1402029 25646307 PMC4340715

[133] Grützke B Hucke S Gross CC Herold MVB Posevitz-Fejfar A Wildemann BT. Fingolimod treatment promotes regulatory phenotype and function of B cells. Ann Clin Transl Neurol. (2015) 2:119–30. 10.1002/acn3.155 25750917 PMC4338953

[134] Hardy TA. Regulatory B cells: the key to post-alemtuzumab rebound phenomena in multiple sclerosis? Mult Scler. (2019) 88:1945–6. 10.1177/1352458518810258 31749413

[135] Wu Q Mills EA Wang Q Dowling CA Fisher C Kirch B. Siponimod enriches regulatory T and B lymphocytes in secondary progressive multiple sclerosis. JCI Insight. (2020) 5:e134251. 10.1172/jci.insight.134251 31935197 PMC7098784

[136] Kappos L Hartung HP Freedman MS Boyko A Radü EW Mikol DD. Atacicept in multiple sclerosis (ATAMS): a randomised, placebo-controlled, double-blind, phase 2 trial. Lancet Neurol. (2014) 13:353–63. 10.1016/S1474-4422(14)70028-6 24613349

[137] Bennett JL Cabre P Carroll W Jacob A Jarius S Lana-peixoto M. International consensus diagnostic criteria for neuromyelitis optica spectrum disorders. Neurology. (2015) 85:177–89. 10.1212/WNL.0000000000001729 26092914 PMC4515040

[138] Lennon VA Wingerchuk DM Kryzer TJ Pittock SJ Lucchinetti CF Fujihara K. A serum autoantibody marker of neuromyelitis optica: distinction from multiple sclerosis. Lancet (London, England). (2004) 364:2106–12. 10.1016/S0140-6736(04)17551-X 15589308

[139] Saadoun S Waters P Bell BA Vincent A Verkman AS Papadopoulos MC. Intra-cerebral injection of neuromyelitis optica immunoglobulin G and human complement produces neuromyelitis optica lesions in mice. Brain. (2010) 133:349–61. 10.1093/brain/awp309 20047900 PMC2822632

[140] Cotzomi E Stathopoulos P Lee CS Ritchie AM Soltys JN Delmotte FR. Early B cell tolerance defects in neuromyelitis optica favour anti-AQP4 autoantibody production. Brain. (2019) 142:1598–615. 10.1093/brain/awz106 31056665 PMC6536857

[141] Shaygannejad V Fayyazi E Badihian S Mirmosayyeb O Manouchehri N Ashtari F. Long-term tolerability, safety and efficacy of rituximab in neuromyelitis optica spectrum disorder: a prospective study. J Neurol. (2019) 266:642–50. 10.1007/s00415-019-09180-9 30635724

[142] Quan C Yu H Qiao J Xiao B Zhao G Wu Z. Impaired regulatory function and enhanced intrathecal activation of B cells in neuromyelitis optica: distinct from multiple sclerosis. Mult Scler. (2013) 19:289–98. 10.1177/1352458512454771 22864301

[143] Han J Sun L Wang Z Fan X Wang L Song Y-Y. Circulating regulatory B cell subsets in patients with neuromyelitis optica spectrum disorders. Neurol Sci. (2017) 38:1205–12. 10.1007/s10072-017-2932-7 28389940

[144] Araki M Matsuoka T Miyamoto K Kusunoki S Okamoto T Murata M. Efficacy of the anti-IL-6 receptor antibody tocilizumab in neuromyelitis optica: a pilot study. Neurology. (2014) 82:1302–6. 10.1212/WNL.0000000000000317 24634453 PMC4001188

[145] Assier E Boissier M-C Dayer J-M. Interleukin-6: from identification of the cytokine to development of targeted treatments. Joint Bone Spine. (2010) 77:532–6. 10.1016/j.jbspin.2010.07.007 20869898

[146] Höftberger R Guo Y Flanagan EP Lopez-Chiriboga AS Endmayr V Hochmeister S. The pathology of central nervous system inflammatory demyelinating disease accompanying myelin oligodendrocyte glycoprotein autoantibody. Acta Neuropathol. (2020) 139:875–92. 10.1007/s00401-020-02132-y 32048003 PMC7181560

[147] Loos J Pfeuffer S Pape K Ruck T Luessi F Spreer A. MOG encephalomyelitis: distinct clinical, MRI and CSF features in patients with longitudinal extensive transverse myelitis as first clinical presentation. J Neurol. (2020) 267:1632–42. 10.1007/s00415-020-09755-x 32055995 PMC7293681

[148] Li X Wang L Zhou L ZhangBao J Miao MZ Lu C. The imbalance between regulatory and memory B cells accompanied by an increased number of circulating T-follicular helper cells in MOG–antibody-associated demyelination. Mult Scler Relat Disord. (2019) 36:101397. 10.1016/j.msard.2019.101397 31546225

[149] Joly MS Martin RP Mitra-Kaushik S Phillips L D'Angona A Richards SM. Transient low-dose methotrexate generates B regulatory cells that mediate antigen-specific tolerance to alglucosidase alfa. J Immunol. (2014) 193:3947–58. 10.4049/jimmunol.1303326 25210119

[150] Calderón-Gómez E Lampropoulou V Shen P Neves P Roch T Stervbo U. Reprogrammed quiescent B cells provide an effective cellular therapy against chronic experimental autoimmune encephalomyelitis. Eur J Immunol. (2011) 41:1696–708. 10.1002/eji.201041041 21469107 PMC3431508

